# The brain in three crustaceans from cavernous darkness

**DOI:** 10.1186/s12868-015-0138-6

**Published:** 2015-04-07

**Authors:** Martin EJ Stegner, Torben Stemme, Thomas M Iliffe, Stefan Richter, Christian S Wirkner

**Affiliations:** Allgemeine und Spezielle Zoologie, Institut für Biowissenschaften, Universität Rostock, Universitätsplatz 2, 18055 Rostock, Germany; Division of Cell Biology, University of Veterinary Medicine Hannover, Bischhofsholer Damm 15, 30173 Hannover, Germany; Department of Marine Biology, Texas A&M University at Galveston, 200 Seawolf Parkway, Galveston, TX 77553 USA

**Keywords:** Optic neuropil, Central complex, Hemiellipsoid body, Neurophylogeny, Olfactory globular tract, Olfactory lobe, Ventral nerve cord, Mechanosensory neuropil

## Abstract

**Background:**

While a number of neuroanatomical studies in other malacostracan taxa have recently contributed to the reconstruction of the malacostracan ground pattern, little is known about the nervous system in the three enigmatic blind groups of peracarids from relict habitats, Thermosbaenacea, Spelaeogriphacea, and Mictocarididae. This first detailed description of the brain in a representative of each taxon is largely based on a combination of serial semi-thin sectioning and computer-aided 3D-reconstructions. In addition, the mictocaridid *Mictocaris halope* was studied with a combination of immunolabeling (tubulin, nuclear counter-stains) and confocal laser scanning microscopy, addressing also the ventral nerve cord.

**Results:**

Adjacent to the terminal medulla, all three representatives exhibit a distal protocerebral neuropil, which is reminiscent of the lobula in other Malacostraca, but also allows for an alternative interpretation in *M. halope* and the thermosbaenacean *Tethysbaena argentarii*. A central complex occurs in all three taxa, most distinctively in the spelaeogriphacean *Spelaeogriphus lepidops*. The deutocerebral olfactory lobe in *M. halope* and *S. lepidops* is large. The comparably smaller olfactory lobe in *T. argentarii* appears to be associated with a unique additional deutocerebral neuropil. A small hemiellipsoid body exists only in the protocerebrum of *T. argentarii*. Distinctive mechanosensory neuropils corresponding to other malacostracans are missing.

**Conclusions:**

The considerable reduction of the optic lobe in the studied taxa is higher than in any other blind malacostracan. The large size of deutocerebral olfactory centers implies an important role of the olfactory sense. The presence of a distinctive central complex in the blind *S. lepidops* adds further support to a central-coordinating over a visual function of this structure. The lack of a hemiellipsoid body in *M. halope* and *S. lepidops* suggests that their terminal medulla takes over the function of a second order olfactory center completely, as in some other peracarids. The reduction of the optic lobe and hemiellipsoid body is suggested to have occurred several times independently within Peracarida. The missing optic sense in the studied taxa is not correlated with an emphasized mechanosense.

## Background

### General aspects

**Figure 1:**
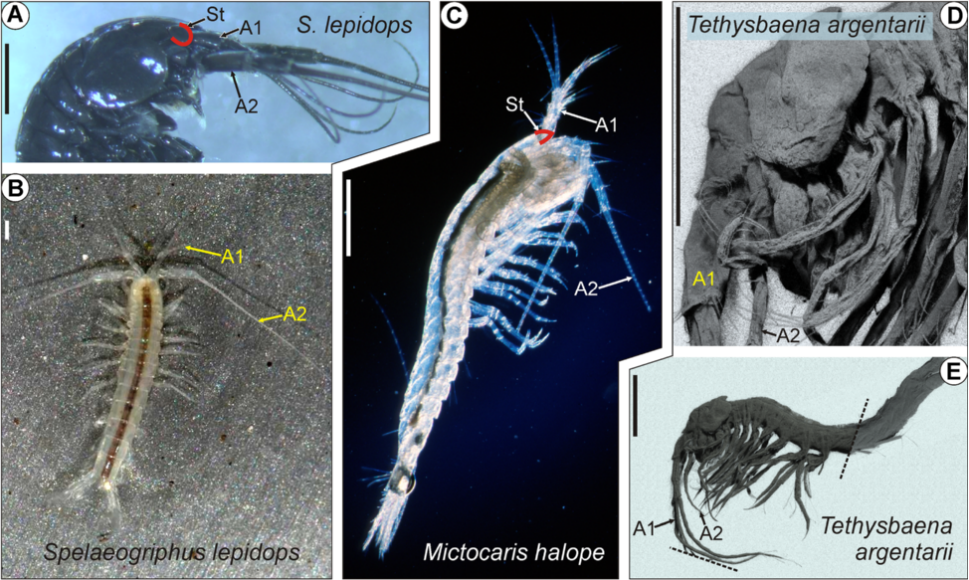
**Overview of the three blind peracarids studied. A**, **B**: *Spelaeogriphus lepidops*, (Spelaeogriphacea) exhibits an eyeless rudimentary eyestalk **(A)**, a slender antenna 1 and a prominent antenna 2 (dark hue of specimen in **A** due to artificial staining). **B**: Courtesy by G. Giribet. **C**: *Mictocaris halope* (Mictocarididae) exhibits an eyeless rudimentary eyestalk, and a prominent antenna 1 and 2. **D**, **E**: Confocal micrographs of cuticular autofluorescence. In contrast to the other species, the antenna 1 of *Tethysbaena argentarii* is more prominent than the slender antenna 2. An eyestalk is absent. Scale bars: 0.5 mm.

Peracarida are one of the most species-rich taxa of Malacostraca. While more familiar peracarid subtaxa, such as Isopoda or Amphipoda, show an impressive morphological disparity which allowed them to adapt to diverse ecosystems, little is known about the three subtaxa investigated here (see Figure 1), all of which occur in relict habitats that are accessible only with considerable difficulty and risk. Mictocarididae and Spelaeogriphacea are found in a few anchialine or fresh-water caves that are punctually distributed over erstwhile Gondwana, while Thermosbaenacea live in thermal springs as well as in anchialine and fresh-water caves.

While the monophyly of Spelaeogriphacea and Thermosbaenacea is well-supported [1], the situation in Mictocarididae is less clear. Some authors assumed monophyletic Mictacea, comprising Mictocarididae and Hirsutiidae ([2], see also [3,4]). Others suggested that Mictocarididae are a sister group to Spelaeogriphacea and assigned Hirsutiidae to the separate taxon Bochusacea [5,6]. Here the abbreviation MST sums up the three taxa Mictocarididae, Spelaeogriphacea, and Thermosbaenacea without any phylogenetic implications―well aware that Mictacea, Spelaeogriphacea, and Thermosbaenacea have been suggested to form a monophylum nested within Peracarida by some authors [7,8].

### Neuroanatomy in Malacostraca

Although neuroanatomical studies within Malacostraca have typically concentrated on Decapoda (e.g., [9-21]), there is also information available from non-decapod taxa such as Leptostraca [22], Stomatopoda [23], Anaspidacea and Euphausiacea [24] as well as most peracarid subtaxa including Mysida [25,26], Lophogastrida, Tanaidacea [27], Cumacea [28,29], Isopoda [29-32], and Amphipoda [33,34]. Despite this considerable taxonomic range, a comprehensive picture of malacostracan brain evolution remains impossible because many studies did not cover the complete brain, but rather focused on chosen structures of phylogenetic or physiological interest, such as the optic system, the olfactory system or the central complex. Our study establishes the first comprehensive description of the brain in MST. Apart from a few overviews of the brain in MST [35,36], these peracarid taxa have not been investigated so far.

### Adaptations to darkness

All representatives of MST live in the darkness and lack eyes. Eyestalks are still present in Mictocarididae [37], Spelaeogriphacea [38], and in the thermosbaenacean subtaxon Halosbaenidae [39], but are absent in other thermosbaenaceans [39-41] and Hirsutiidae [37]. Various crustacean neuroanatomists addressed the correlations between lifestyle and adaptations of the visual (e.g., [10,30,42,43]), olfactory (e.g., [13,19-21]), mechanosensory centers (e.g., [31]), or unpaired midline neuropils [43,44]. One focus of this study is to infer whether and how the brain has changed in correlation to reductions of the eyes and eyestalks in MST, i.e., whether optic neuropils have been reduced, and whether the loss of visual input has been compensated by a notable emphasis on the olfactory or mechanosensory pathways or other parts of the nervous system.

### Phylogenetic aspects

The morphology of the brain in MST may furthermore help resolve their controversial phylogenetic positions within Peracarida. The comparison of neural structures had considerable impact on arthropod phylogenetics in recent years (e.g., [15,16,45]). A close relationship between Mictocarididae (Hirsutiidae were excluded from analyses) and Spelaeogriphacea was supported by most studies, but their exact position within Peracarida remains debatable [1,4,8,46,47]. Thermosbaenacea have repeatedly been placed as a sister group to all (remaining) Peracarida [1,48,49]. Other authors placed them in close relationship with Mictacea [8,47,50] or elsewhere within Peracarida [46,51,52].

We investigated one species of each taxon combining serial semi-thin sections with computer-aided 3D-reconstruction, a method well-accepted to reveal the brain’s soma clusters, major nerves, and neuropils (e.g., [20-22,53,54]), but also the relationships of these neural structures to other organ systems within the cephalon [35,36]. In the mictocaridid representative *Mictocaris halope*, we were fortunate to obtain several additional specimens preserved for immunolabeling with an antibody against acetylated α-tubulin and nuclear counterstaining, which allowed for a detailed tracing of neurite tracts and neuropil texture in this species. Our morphological description in all three species investigated adds new characters to the phylogenetic debate and contributes to a more coherent picture of brain evolution within Malacostraca.

## Results

### Note on terminology

**Figure 2:**
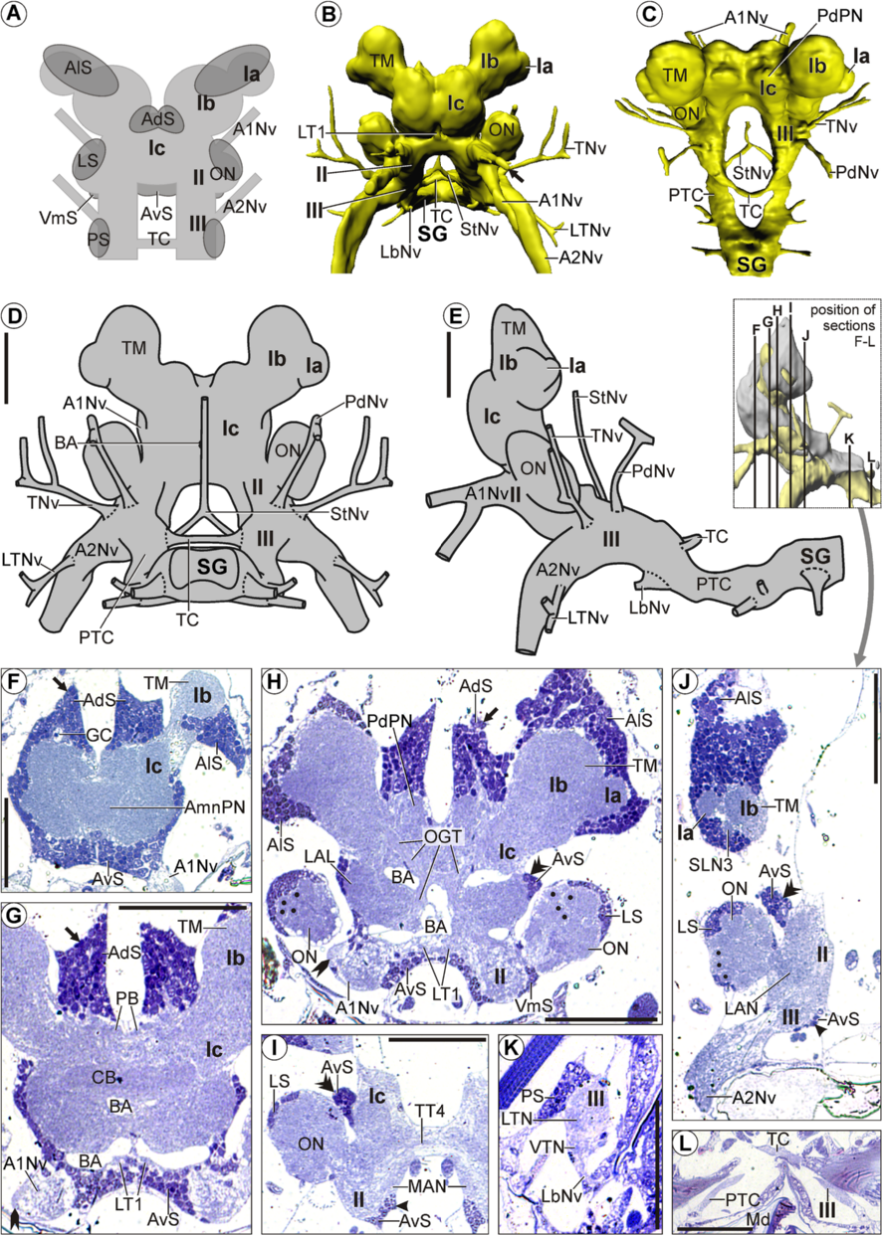
**Morphology of the brain in** ***Mictocaris halope*** **.** Overview and semi-thin sections. **A**: Schematic depiction of our simplified nomenclature for soma clusters. **B**-**E**: Neuropil and nerves without somata. **B**, **C**: 3D-reconstructions in **(B)**: anterior and **(C)**: dorsal view. **D**, **E**: Schematic drawing in **(D)**: posterior and **(E)**: lateral view. **F**-**L**: Transverse semi-thin sections, ordered from anterior to posterior. **F**-**H**: Arrow points at a large dorsal extension of anterodorsal somata (AdS). **G**, **H**: Rocket points at the lateral root of the antenna 1 nerve (A1Nv). **H**: Points mark the olfactory glomeruli in the olfactory lobes (ON). **H**-**J**: Double arrowheads point at the large lateral extensions of lateral somata (LS). **I**-**J**: Simple arrowhead points at a large ventral extension of anteroventral somata (AvS). Scale bars: 50 μm.

We widely apply the neuroanatomical terminology suggested for the decapod brain by Sandeman *et al*. [55], and extend it only where necessary. In comparison to the many soma clusters in Decapoda, for which Sandeman *et al.* [55] suggested a labeling that is widely accepted, the studied representatives of MST feature considerably less soma clusters that are separated from each other by soma-free regions. A soma cluster in this sense may comprise somata from one or more brain regions. We refer to somata in six different brain regions, applying a simple nomenclature schematically explained in Figure 2A: anterolateral somata (AlS) surround the optic lobes and the lateral protocerebrum; anterodorsal somata (AdS) lie dorsally in the median protocerebrum; anteroventral somata (AvS) lie ventrally in the median protocerebrum; lateral somata (LS) lie laterally in the deutocerebrum; ventromedial somata (VmS) lie ventrally in the deutocerebrum; posterior somata (PS) lie laterally and dorsally in the tritocerebrum.

The morphological description of spatially extended structures often requires giving border points and using directional terms, such as ‘run into’ or ‘project anteriorly’. In our work, this does not imply any physiological, ontogenetic, or evolutionary direction. All positions given (such as anterior or dorsal) refer to the body axis. The term ‘medial’ is used, when two paired structures lie near the midline of the animal, whereas the term ‘median’ is used, when an unpaired structure lies directly in the midline of the animal. The identification of blood vessels was based on Wirkner and Richter [35] for *M. halope* and *S. lepidops*, and on Wirkner and Richter [36] for *T. argentarii*.

Sullivan and Beltz [23] pointed out that what is generally referred to as the ‘terminal medulla’ in the malacostracan protocerebrum is rather an assembly of several neuropils than one coherent neuropil. We use ‘terminal medulla’ as an inclusive term for all that neuropil of the lateral protocerebrum which is *not* clearly specified from its surroundings by shape, texture, or spatial separation. The described ‘small lateral neuropils’ of our study are specified by their dense texture and clear spatial separation from the terminal medulla in the periphery of the lateral protocerebrum. In contrast to the terminal medulla, our criteria for the identification of a hemiellipsoid body are its distally convex shape, its dense texture, and its anteromedial position within the lateral protocerebrum, as found in other Malacostraca [22,55].

The brain comprises a number of tracts, which we here divided into vertical (VT), longitudinal (LT), and transverse tracts (TT), and numbered consecutively. If tracts in different species occur in the corresponding position, they received the same label (e.g., LT1 was found in *M. halope* and *S. lepidops*). All vertical and longitudinal tracts described occur in pairs. Transverse tracts are unpaired and midline-spanning if not otherwise noted.

### Major results

As in other Peracarida, there are no signs of a nauplius eye or nauplius eye-related neural structures in MST. In all three species studied, the brain is a syncerebrum composed of protocerebrum, deutocerebrum, and tritocerebrum. The protocerebrum is divided into a distal protocerebral neuropil (which is here labeled ‘Ia’ due to its unresolved homology relationships), the lateral protocerebrum (Ib), and the unpaired median protocerebrum (Ic). A distinctive hemiellipsoid body occurs only in the lateral protocerebrum of *T. argentarii*. The deutocerebrum (II) gives rise to the antenna 1 nerve and includes the olfactory lobe. The tritocerebrum (III) gives rise to the antenna 2 nerve. Additional nerves arise mainly from the deuto- and tritocerebrum. One post-esophageal commissure interconnects the paired halves of the tritocerebrum transversely. On each side, the tritocerebrum is connected to the posterior-next ganglion by a soma-free connective. *S. lepidops* features a separate mandibular ganglion, while in *M. halope* and *T. argentarii,* a subesophageal ganglion is composed of several neuromeres. Somata are arranged in only a few, often large clusters around the brain's neuropil. Soma clusters differ interspecifically in size and shape (see above for terminology of these soma clusters). Each species features a number of neuropils, the most prominent of which are the central body and protocerebral bridge. Proto-, deuto-, and tritocerebrum are arranged along a neuraxis which deflects from the body axis anteriorly and shows a different course in each species.

### *Mictocaris halope* (Mictocarididae)

#### General aspects

*M. halope* lacks compound eyes. The pyriform eye stalk inserts anteriorly in the cephalon (position indicated in Figure 1C). Antenna 1 exhibits a prominent 3-segmental peduncle bearing a 4-segmented inner and an 8-segmented outer flagellum ([37]; see also Figure 1C). Each of the five distalmost segments of the outer flagellum bears one very long aesthetasc ([37]; see also Figure 1C). Antenna 2 exhibits a prominent 4-segmented peduncle, which bears a large scale on its second segment and, distally, a 35-segmented flagellum [37]. Bowman and Iliffe [37] described several loosely arranged setae both on antenna 1 and 2.

**Figure 3:**
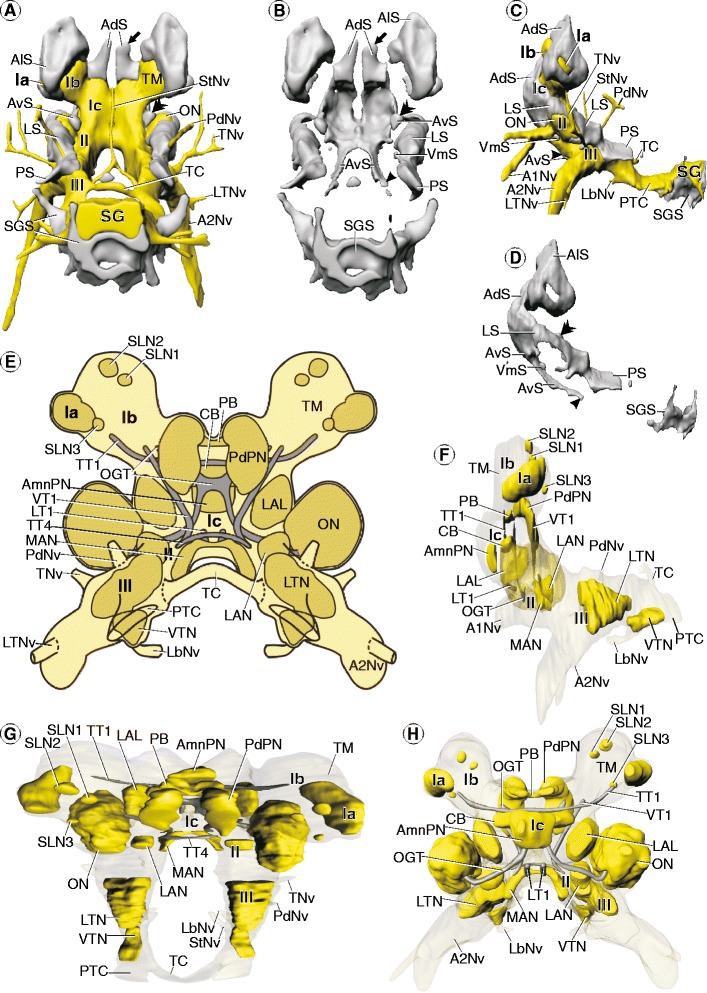
**Morphology of the brain in** ***Mictocaris halope*** **.** Soma clusters, neuropil, and internal structure – click on A and H for interactive 3D models. **A**,**B**: Soma clusters (grey) in posterior view **(A)**: with neuropil (yellow) and **(B)**: without neuropil. **C**, **D**: Soma clusters in lateral view **(C)**: with and **(D)**: without neuropil. **A**-**D**: Arrows mark a dorsal extension of anterodorsal somata (AdS). Double arrowheads mark a lateral extension of lateral somata (LS). Simple arrowhead marks a ventral extension of anteroventral somata. **E**: Schematic drawing of neuropils (dark yellow) and tracts (grey) in posterior view. **F**-**H**: 3D-reconstructions of neuropils and tracts in **(F)**: lateral, **(G)**: dorsal, and **(H)**: anterior view. Neuropils are in yellow; tracts are in grey. **F**: Olfactory lobe (ON) is shown semitransparent. Brain width about 200 μm (perspective view).

**Figure 4:**
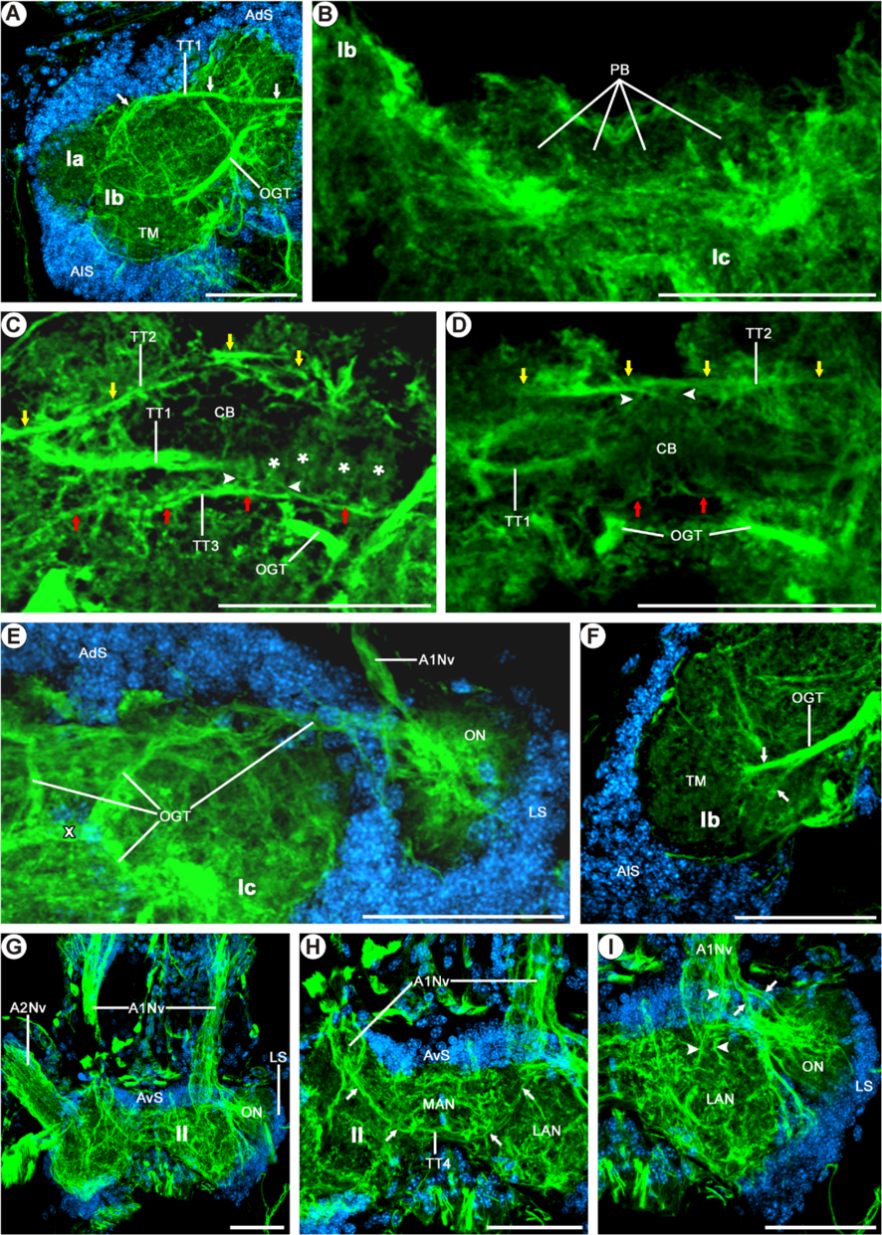
**Morphology of the brain in** ***Mictocaris halope*** **.** Acetylated α-tubulin immunoreactivity and nuclear counterstaining. **A**-**I**: Dorsal view on confocal laser-scans of horizontal vibratome sections (50 μm) labeled for acetylated α-tubulin immunoreactivity (green) and nuclear marker (blue). **A**: Overview of distal protocerebral neuropil (Ia) and lateral protocerebrum (Ib). **B**: The neuropilar subunits of the protocerebral bridge (PB) are visible as a negative imprint at the anterior part of the median protocerebrum (Ic). **C**, **D**: The central body (CB) is subdivided into several elongated or spheroidal compartments (asterisks), which are connected posteriorly via numerous fine neurites (arrowheads in **C**) to the 3^rd^ transverse tract (TT3, red arrows). The 2^nd^ transverse tract (TT2, yellow arrows) passes the CB anteriorly, sending fine processes into the neuropil (arrowheads in **D)**. The 1^st^ transverse tract (TT1) lies dorsal to the CB. **E**, **F**: The paired branches of the OGT form a characteristic chiasm (X in E) in the center of the median protocerebrum (Ic) and connect the olfactory neuropil (ON) to the lateral protocerebrum (Ib). Before reaching the target structure in the medial part of the lateral protocerebrum, the OGT splits into two neurite bundles (arrows in **F**). **G**: Overview of the deutocerebrum (II). **H**: Higher magnification of the medial part of the deutocerebrum (II). The 4^th^ transverse tract (TT4) seems to originate in the nerves of the antenna 1 (A1Nv), forming a deutocerebral commissure (arrows). **I**: The antenna 1 nerves (A1Nv) enter the deutocerebrum from the anterior direction, each splitting into a thick medial and a slender lateral branch. Several neurite bundles from the medial branch project into the lateral deutocerebral neuropil (arrowheads), while others constitute to the 4^th^ transverse tract (TT4) (arrows in **H**). The lateral branch innervates the olfactory neuropil (arrows). Scale bars: 50 μm.

The distal protocerebral neuropil (Ia) is situated directly lateral to the lateral protocerebrum (Ib, Figures 2B-E, 3H, 4A), which is adjoined by the median protocerebrum (Ic) ventromedially (Figures 2B-H, 3A,C, 4A). In comparison to the other species, the olfactory lobe is relatively large and ellipsoid, protruding laterally from the rest of the deutocerebrum (Figures 2B-E,H-J, 3A,C, 4E,G,I). In lateral aspect, the lateral protocerebrum is situated posterodorsal to the median protocerebrum, and the latter anterodorsal to the deutocerebrum (Figures 2E, 3C). The neuraxis flattens posteriorly to take on the anteroposterior course of the ventral nerve cord (III, SG; Figures 2E, 3C). The unpaired brain artery (BA) enters the median protocerebrum from the posterior direction (Figure 2D). The artery first splits into an upper and a lower branch (Figure 2G), each of which bifurcates into one pair of sub-branches leaving the median protocerebrum laterally (Figure 2H).

#### Soma clusters

The brain in *M. halope* features three pairs of soma clusters and one unpaired soma cluster (i.e., altogether seven clusters).

The first paired soma cluster is composed of anterolateral somata (AlS), which cover the distal protocerebral neuropil and lateral protocerebrum dorsally, ventrolaterally, and posteriorly (Figures 2F,H,J, 3A,C, 4A,F). It is dorsally extended (AlS; Figures 2J, 3A-D).

The second paired soma cluster consists both of lateral somata (LS) covering the olfactory lobes anteriorly, dorsolaterally, and posteriorly (Figures 2H-K, 3A-D, 4E,G,I) and of posterior somata (PS) which are situated dorsolaterally in the tritocerebrum (Figures 2K, 3A-D). The posterior somata form a short lobe-like extension pointing posterolaterally (Figures 2K, 3A-D).

The third paired soma cluster is minute, only comprising a few ventromedial somata (VmS) that lie ventrally in the deutocerebrum between the olfactory lobe and the root of the antenna 1 nerve (A1Nv; Figures 2H, 3B-D).

The unpaired soma cluster comprises anterodorsal somata (AdS) and anteroventral somata (AvS) and extends from the dorsal (Figures 2F-H, 3A,B) over the anterior (Figure 3B-D, 4A,E) to the ventral region of the median protocerebrum (Figures 2F-J, 3B-D). On each side, the soma cluster shows a long dorsal extension (AdS, arrows; Figures 2F-H, 3A,B), a lateral extension (AvS, double arrowheads; Figures 2H-J, 3A,B,D), and a ventral extension (AvS, simple arrowheads; Figures 2I,J, 3B-D). Each dorsal extension contains a small globular cavity reminiscent of the Bellonci organ in other crustaceans (GC; Figure 2F). The lateral extension extends posteriorly along the lateral side of the median protocerebrum as far as the olfactory lobe (Figures 2H-J, 3A). The ventral extension extends posteriorly along the ventromedial side of the deutocerebrum (II) as far as the tritocerebrum (III; Figures 2I,J, 3B-D).

#### Neuropils

##### Distal protocerebral neuropil (Ia)

The distal protocerebral neuropil (Ia) has a spheroidal shape and appears as a uniformly structured neuropil that is in part confluent with the terminal medulla (Figures 2B-E,H,J, 3E-H, 4A). In our histological and immunocytochemical preparations, we detected neither any nerve nor tract extending from the distal protocerebral neuropil into the eyestalk.

##### Lateral protocerebrum (Ib)

On each side, the lateral protocerebrum comprises, on the one hand, the large terminal medulla (TM; Figures 2B-H,J, 3A,C,E-H, 4A,F) and, on the other hand, three small lateral neuropils (SLN1-3 in Figure 3E-H; see also SLN3 in Figure 2J). The terminal medulla is evenly textured, with subcompartments that are hard to distinguish. We could not identify a hemiellipsoid body. The 1^st^ small lateral neuropil lies dorsomedially, the 2^nd^ dorsally (Figure 3E-H), and the 3^rd^ posteriorly in the periphery of the lateral protocerebrum (Figures 2J, 3E-H).

##### Median protocerebrum (Ic)

The unpaired anteromedian protocerebral neuropil (AmnPN) lies anteriorly in the median protocerebrum (Figures 2F, 3E-H). Posteriorly, it is confluent with its surroundings. The unpaired cigar-shaped central body (CB) lies horizontally across the center of the median protocerebrum and has a comparatively dense texture (Figures 2G, 3E,F,H, 4C,D). Acetylated α-tubulin immunoreactivity reveals several spheroidal subcompartments within the central body (asterisks; Figure 4C). The unpaired protocerebral bridge (PB) is situated dorsally in the median protocerebrum (Ic) and consists of at least four spheroidal subunits (here counting both body sides together) with a comparatively dense texture (Figures 2G, 3E-H). These compartments are visible as a negative imprint in the immunocytochemical preparations (Figure 4B). On each side, the posterodorsal protocerebral neuropil (PdPN) extends from the dorsal to the posterior region of the median protocerebrum (Figures 2C,H, 3E-H). The lateral region of the median protocerebrum shows a comparatively dense texture and is here interpreted as the lateral accessory lobe (LAL; Figures 2H, 3E-H). Medially, the lateral accessory lobe is confluent with its surroundings.

##### Deutocerebrum (II)

The lateral antenna 1 neuropil (LAN) lies laterally in the deutocerebrum and medial to the olfactory lobe (Figures 2J, 3E-H, 4G-I). The large and ellipsoid olfactory lobe (ON; Figures 2H-J, 3A,C,E-H, 4E,G,I) is composed of a number of nearly spheroidal olfactory glomeruli (points; Figure 2H,J). The median antenna 1 neuropil (MAN) spans horizontally across the median region of the deutocerebrum (Figures 2I, 3E-H, 4H). Since its halves are only connected by a tract, the median antenna 1 neuropil in *M. halope* has to be considered as paired.

##### Tritocerebrum (III)

The lateral tritocerebral neuropil (LTN) lies laterally in the tritocerebrum (Figures 2K, 3E-H). Posteriorly, it is confluent with its surroundings. The ventral tritocerebral neuropil (VTN) extends longitudinally along the ventral side of the tritocerebrum (III; Figures 2K, 3E-H). It is situated directly posterior to the nerve root of the labral nerve (Figures 2K, 3E-H). Posteriorly, it is confluent with its surroundings. The posterior region of the tritocerebrum is dorsoventrally flattened (III; Figure 2L) and confluent with the post-tritocerebral connectives.

#### Tracts

The olfactory globular tract (OGT) connects the center of the olfactory lobe with the terminal medulla (Figures 2H, 3E,F,H, 4E,F). Within the terminal medulla, the olfactory globular tract splits into two conspicuous branches, one terminating medially and the other anterolaterally in the undifferentiated terminal medulla (arrows; Figure 4F). The olfactory globular tracts of both body sides decussate posterior to the central body (Figures 2H, 3E, 4E). The 1^st^ vertical tract (VT1) extends from the posteroventral region of the terminal medulla to the deutocerebrum in the medioventral direction (Figure 3E,F,H). The 1^st^ transverse tract (TT1) starts near the distal protocerebral neuropil in the ventral region of the terminal medulla, from there extending to the other side of the body (Figures 3E-H, 4A,C). On its way, it passes the central body dorsally, but we could not identify whether it is associated with it (Figures 3E,F,H, 4C). The second and third transverse tracts (TT2, TT3), identified by immunocytochemical staining, span across the median protocerebrum. Each of these tracts is associated with the central body, one passing it anteriorly (TT2) and one posteriorly (TT3, Figure 4C,D). The anterior of these tracts sends neurites into the central body where the neurites form a conspicuous chiasm with their counterparts from the other body side. The posterior tract contributes neurites to the subcompartments of the central body (arrowheads in Figure 4C). The 4^th^ transverse tract (TT4) interconnects the anteromedial region of the deutocerebrum of both body sides and is slightly bent up in the middle (Figures 2I, 3E,G, 4H). Based on acetylated α-tubulin staining, this tract originates near the root of the antenna 1 nerve (arrows; Figure 4H). On each side, the 1^st^ longitudinal tract (LT1) extends along the ventral side of the median protocerebrum (Ic) in the posteroventral direction (Figures 2G,H, 3E,F,H). For a short section, the tract leaves the surrounding neuropil of the median protocerebrum and is bordered only by a lateral branch of the brain artery and anteroventral somata (Figure 2G,H). The tritocerebral commissure (TC) interconnects the opposite halves of the tritocerebrum transversely (Figures 2B-E,L, 3A,C,E-G). It is slightly bent up in the middle. On each side, the dorsoventrally flattened post-tritocerebral connective (PTC) connects the tritocerebrum to the subesophageal ganglion (Figures 2C-E,L, 3C).

#### Nerves

On each side, the antenna 1 nerve (A1Nv) enters the deutocerebrum from the anterior direction (Figures 2B,E-H, 3C,F, 4E,G-I), thereby bifurcating into a thick medial and a thin lateral root (Figure 4I). The medial root of the antenna 1 nerve (Figures 2H, 4I) enters the lateral antenna 1 neuropil (Figures 2J, 3F). Neurites in the lateral root are arranged more densely (rocket in Figure 2G,H). Immunolabeling reveals that the lateral root splits again into two neurite bundles (arrows in Figure 4I). The more lateral bundle proceeds directly into the center of the olfactory lobe (ON; Figures 2H, 4I), while the more medial bundle forms a decussation with the olfactory globular tract directly medial to the olfactory lobe (Figure 4I). Distally, each antenna 1 nerve soon splits into several small branches (e.g., Figure 2B, one branch shown by arrow). The prominent antenna 2 nerve (A2Nv) enters the tritocerebrum from the anteroventral direction (Figures 2B,D,E,J, 3A,C,F,H, 4G). In its proximal region, each antenna 2 nerve is entered by a lateral tritocerebral nerve (LTNv; Figures 2B,D,E, 3A,C,E). The tegumentary nerve (TNv) enters the tritocerebrum laterally (Figures 2B-E, 3A,C). Each tegumentary nerve is distally split into three branches (Figures 2B,D, 3A), all of which extend to the body wall. The posterodorsal nerve (PdNv) enters the tritocerebrum from the posterodorsal direction (Figures 2C-E, 3A,C). On its way, it penetrates the described soma cluster which is composed of lateral and posterior somata (Figure 3A,C). The labral nerve (LbNv) enters the posteroventral region of the tritocerebrum from the anteroventral direction (Figures 2B,E,K, 3C). The stomatogastric nerve (StNv) enters the ventromedial region of the tritocerebrum from the anteromedial direction (Figure 2B,C). The stomatogastric nerves from both body sides unite in the midline to form a single unpaired nerve in front of the esophagus (Figures 2B-D, 3A,C). This single nerve extends dorsally (Figures 2D,E, 3A,C) along the frontal side of the gut.

#### Anatomy of the ventral nerve cord

**Figure 5:**
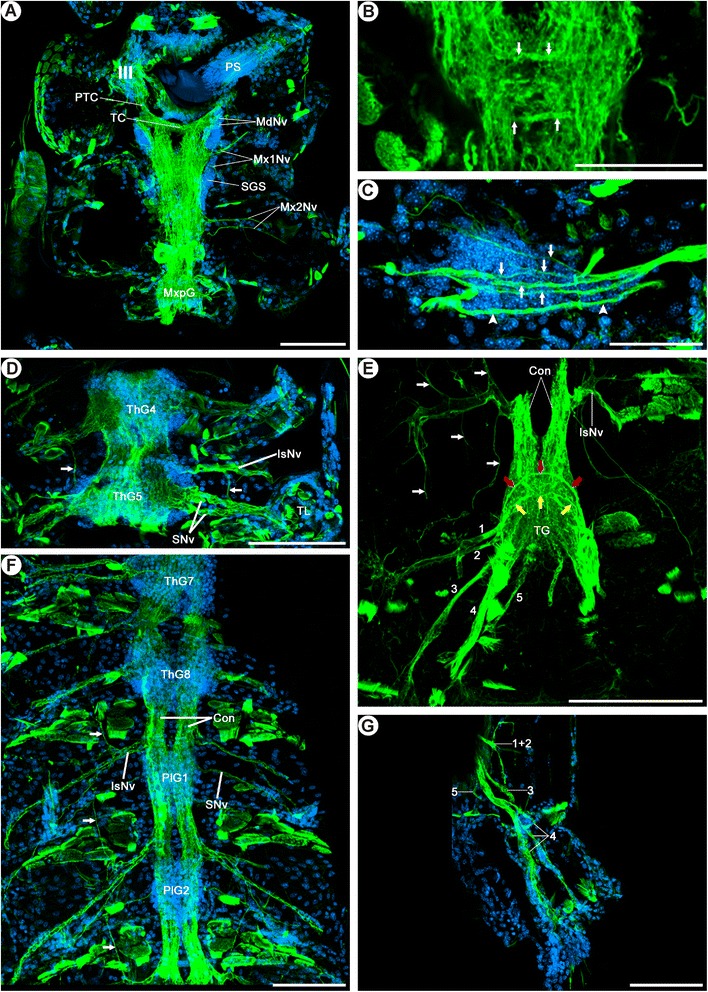
**Morphology of the ventral nerve cord in** ***Mictocaris halope*** **. Acetylated α-tubulin immunoreactivity and nuclear counterstaining. A**-**G**: Dorsal view on confocal laser-scans of horizontal vibratome sections (50 μm) labeled for acetylated α-tubulin immunoreactivity (green) and nuclear marker (blue). **A**: The tritocerebrum (III) is connected posteriorly to the subesophageal ganglion (SG) via the posttritocerebral connectives (PTC). The SG comprises the mandibular, maxillular, and maxillar neuromeres. **B**: Close-up of the maxillular neuromere. Two thick commissure-like neurite bundles (arrows) link the hemiganglia. **C**: The maxillular nerves project laterally into the appendage. The anterior nerve splits into several branches (arrows), whereas the more posterior nerve remains unbranched (arrowheads). **D**: The 2^nd^-8^th^ thoracic ganglia (ThG2-8) are associated with two segmental nerves (SNv), which project laterally into the thoracic limbs (TL) (exemplarily shown for ThG4-5). Furthermore, an intersegmental nerve (IsNv) leaves the connectives laterally. Paired longitudinal lateral neurite bundles are visible parallel to the connectives (arrows). **E**: As in the thorax, an intersegmental nerve (IsNv) leaves each connective (Con) between pleonic ganglia (PlG) laterally and contributes neurites to the lateral longitudinal neurite bundle (arrows). The main branch of the IsNv projects further posterolaterally. One segmental nerve arises from each PlG. **F**: Five nerves (numbers 1-5) extend from the terminal ganglion (TG). Nerves 1 and 2 project in the lateral direction, whereas nerves 3 to 5 extend more posterolaterally. Two commissure-like neurite bundles connect both hemispheres of the TG (red and yellow arrows). The anterior commissure-like neurite bundle (red arrows) is associated with nerves 1 and 2, the posterior commissure-like neurite bundle (yellow arrows) with the nerves 3 and 4. An intersegmental nerve (IsNv) leaves the connectives anterior to the TG and splits into several fine branches innervating the periphery (white arrows). **G**: Nerve 4 extends into the uropods, where it splits into several branches. Scale bars: 100 μm.

The ventral nerve cord of *Mictocaris halope* is composed of segmental ganglia which are interconnected by pairs of soma-free longitudinal connectives (Figure 5). The mandibular, maxillular, and maxillar neuromeres together form a subesophageal ganglion, while each thoracic and pleonic segment features a separate ganglion. The somata of each ganglion are arranged in one midline-spanning cortex, with most somata situated ventrolaterally in the ganglion. The segmental neuropil of both sides is confluent in the midline of each ganglion. Thus, true (free) commissures do not exist in *M. halope* (Figure 5).

##### Subesophageal ganglion

The subesophageal ganglion features one large soma cortex, so that soma-free connectives between its three neuromeres are missing (Figure 5A). A pair of connectives links the subesophageal ganglion anteriorly to the tritocerebrum and posteriorly to the (first thoracic) maxillipedal ganglion (Figure 5A), respectively. Laterally, each of the three hemineuromeres of the subesophageal ganglion gives rise to an anterior and a posterior appendage nerve; both nerves projecting straight laterally into their appendage (Figure 5A). The anterior nerve soon splits into several branches (arrows; Figure 5C), whereas the more posterior nerve remains unbranched (arrowheads; Figure 5C). Other (e.g., intersegmental) nerves in the subesophageal region could not be detected. The mandibular and maxillular neuropils are fused. The maxillar neuropil is set off from the latter by a very short connective-like longitudinal neurite bundle. Both the mandibular/maxillular and the maxillar neuropil are fused with their counterpart on the other body side and embedded in the soma cortex (Figure 5A). Amongst the numerous neurites crossing the midline, a distinct commissure-like anterior and posterior ‘tract’ (*sensu* [56]) are discernible in each subesophageal neuromere (see e.g., in the maxillular neuromere, Figure 5B).

##### Thoracic ganglia 1 to 8

The first thoracic segment is associated with the maxillipeds. The anatomy of the corresponding ganglion is more similar to the other thoracic ganglia than to the maxillar ganglion in terms of size and segmental nerve arrangement. Two segmental nerves leave each thoracic hemiganglion laterally projecting into the appendages (Figure 5D). These segmental nerves are thicker than the segmental nerves of the subesophageal ganglion. An intersegmental nerve projects from each connective laterally into the periphery, starting with the connective between the (first thoracic) maxillipedal segment and the second thoracic segment (Figure 5D). These intersegmental nerves split into three branches. The main branch extends further in the lateral direction; two more slender branches extend anteriorly and posteriorly, respectively (arrows Figure 5D). Both latter branches are connected to the segmental nerves of the anterior and posterior adjacent segments, forming a lateral longitudinal neurite bundle. The laterally extending main branch of the intersegmental nerve could not be traced farther. In contrast to the subesophageal neuromeres, no distinct transverse tracts could be identified within thoracic ganglia.

##### Pleonic ganglia 1 to 5

Pleonic ganglia are smaller than thoracic ganglia (Figure 5E). Each hemiganglion gives rise to one slender pleopod nerve (Figure 5E). An intersegmental nerve arises laterally from each connective of the pleon and splits immediately into three branches similar to the situation in the thoracic ganglia (Figure 5E), one branch extending posterolaterally, one anteriorly, and one posteriorly. The anterior and posterior branches form, together with the corresponding branches of other segments, a lateral longitudinal neurite bundle (arrows, Figure 5E). The posterolateral branch could be traced into the posteriorly adjacent segment until the lateral border of the body. Thus, we suggest, that this branch of the intersegmental nerve innervates the tegument and/or musculature of the lateral body wall. As in the thorax, distinct transverse tracts within the ganglia could not be observed.

##### Terminal ganglion

The terminal ganglion, situated in the sixth pleomere, is larger than the pleonic ganglia 1 to 5 (Figure 5F). Five nerves arise from each hemiganglion (labeled 1-5; see Figure 5F,G). Nerves 1 and 2 extend laterally. These nerves could not be traced to their destination in the periphery. As they do not project towards the uropod or telson, they may innervate the tegument or musculature of the sixth pleomere. Nerves 3, 4 and 5 extend posterolaterally (Figure 5F). While nerve 4 could be traced into the uropod (Figure 5G), nerves 3 and 5 proceed towards the telson. Besides several neurites that cross the midline, the terminal ganglion features two commissure-like transverse tracts (red and yellow arrows; Figure 5F). The more anterior transverse tract (red arrows; Figure 5F) is closely associated with the nerves 1 and 2, the more posterior transverse tract (yellow arrows; Figure 5F) is closely associated with the nerves 3 and 4. An intersegmental nerve arises from the connective between pleonic ganglion 5 and the terminal ganglion. The main branch gives rise to several fine sub-branches that extend anteriorly and posteriorly to innervate the periphery (white arrows; Figure 5F).

##### Lateral neurite bundle

A pair of lateral longitudinal neurite bundles (arrows, Figure 5D, E) is situated lateral to the ventral nerve cord, extending from the first thoracic through the sixth pleonic segment. As described above, these lateral neurite bundles are supplied by neurites arising anteriorly and posteriorly from the intersegmental nerves. While the lateral neurite bundles in the thorax are additionally associated with the segmental nerves, this is not the case in the pleon. No lateral longitudinal neurite bundles have been observed in the subesophageal region.

### *Spelaeogriphus lepidops* (Spelaeogriphacea)

#### General aspects

*S. lepidops* lacks compound eyes. The elongate, ellipsoid eye stalk inserts anteriorly in the cephalon (position indicated in Figure 1A). Antenna 1 exhibits a prominent 3-segmental peduncle bearing an up to 40-segmented inner and an up to 36-segmented outer flagellum ([38]; see also Figure 1A,B). The distal three-fourths of the outer flagellum exhibit a series of short aesthetascs ([38]; see also Figure 1A,B). Antenna 2 exhibits a prominent 4-segmented peduncle, which bears a small scale on its second segment and, distally, an about 70-segmented flagellum [38]. Gordon [38] described fields of conical papilla on antenna 1 and rows of setae both on antenna 1 and 2, but found no statocysts.

**Figure 6:**
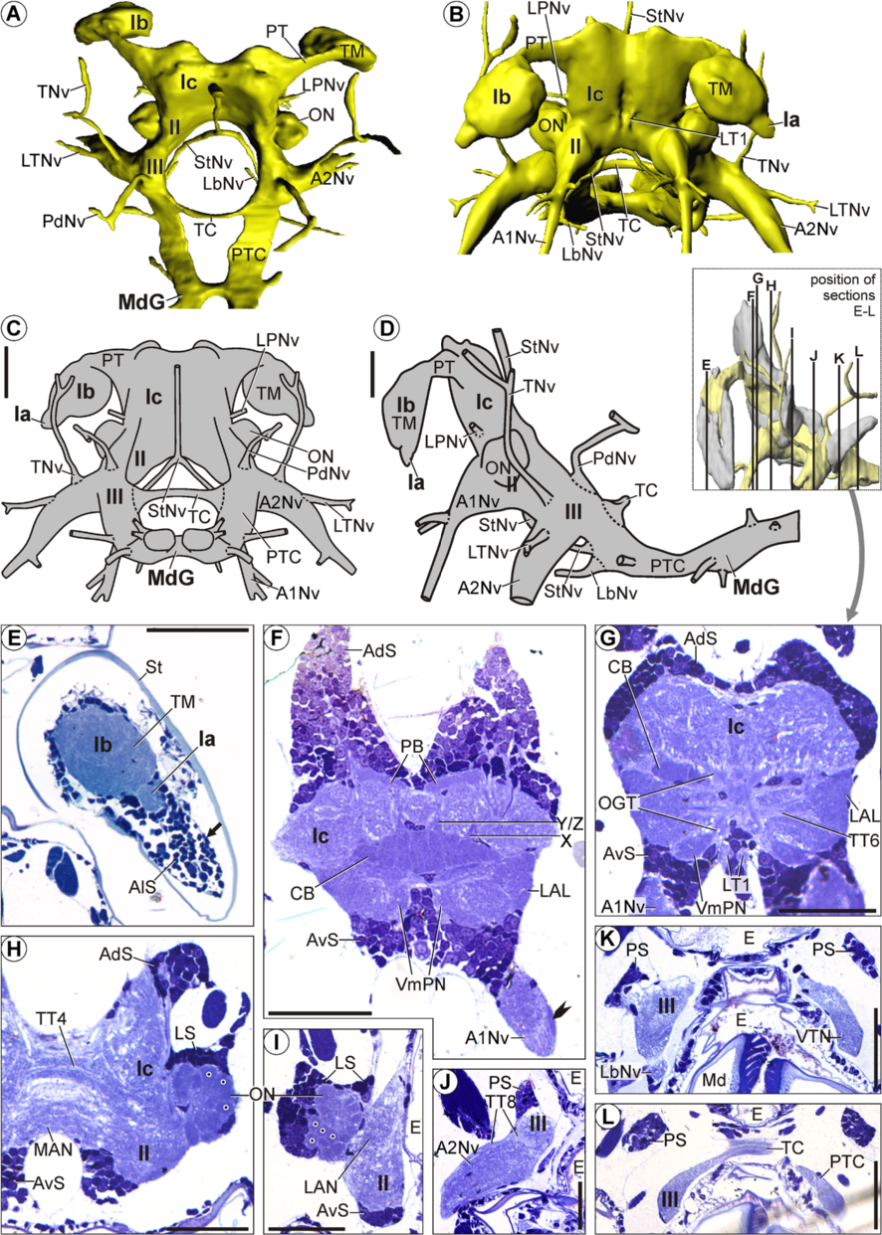
**Morphology of the brain in** ***Spelaeogriphus lepidops*** **.** Overview and semi-thin sections. **A**-**D**: Neuropil and nerves without somata. **A**, **B**: 3D-reconstructions in **(A)**: dorsal and **(B)**: anterior view. **C**, **D**: Schematic drawing in **(C)**: posterior and **(D)**: lateral view. **E**-**L**: Transverse semi-thin sections, ordered from anterior to posterior. **E**: Arrow points at the large cluster of anterolateral somata (AlS). **F**: Rocket points at the lateral root of the antenna 1 nerve (A1Nv). **H**, **I**: Points mark the olfactory glomeruli in the olfactory lobes (ON). Scale bars: 50 μm.

**Figure 7:**
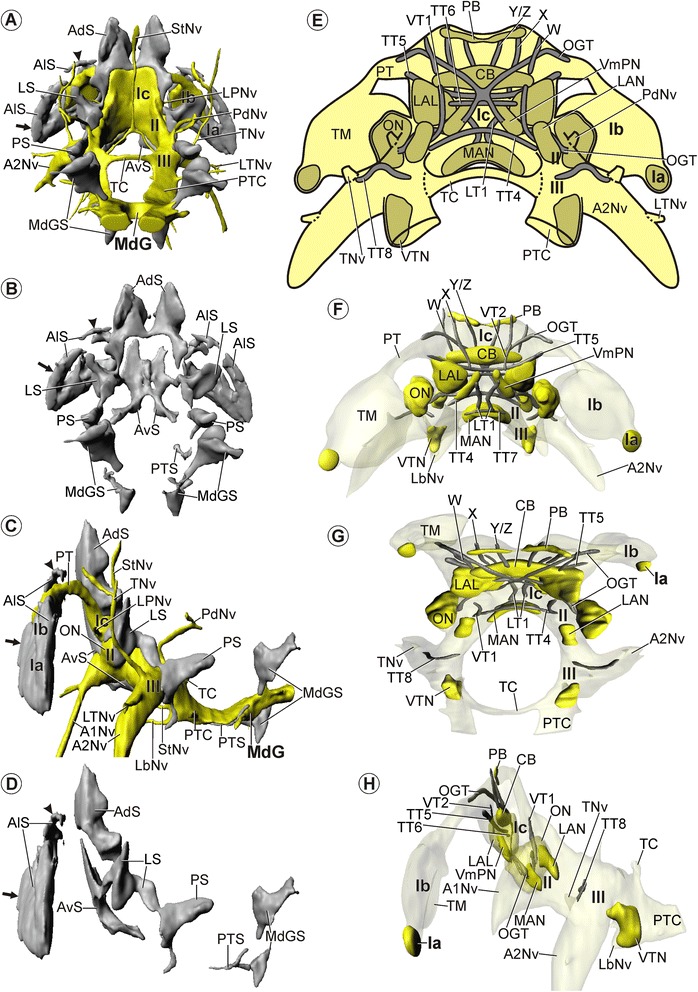
**Morphology of the brain in** ***Spelaeogriphus lepidops*** **.** Soma clusters, neuropil, and internal structure – click on A and F for interactive 3D models. **A**, **B**: Soma clusters (grey) in posterior view **(A)**: with neuropil (yellow) and **(B)**: without neuropil. **C**, **D**: Soma clusters in lateral view **(C)**: with and **(D)**: without neuropil. **A**-**D**: Arrow points at a large cluster and arrowhead at a small cluster of anterolateral somata (AlS). **E**: Schematic drawing of neuropils (dark yellow) and tracts (grey) in posterior view. **F**-**H**: 3D-reconstructions of neuropils and tracts in **(F)**: anterior, **(G)**: dorsal, and **(H)**: lateral view. Neuropils are in yellow; tracts are in grey. **H**: Lateral accessory lobe (LAL) and olfactory lobe (ON) is shown semitransparent. Brain width about 300 μm.

The distal protocerebral neuropil (Ia) and the lateral protocerebrum (Ib; Figure 6B,C,D) are located in the eyestalk (St; Figure 6E) and connected to the median protocerebrum (Ic) via the protocerebral tract (PT; Figures 6A-D, 7C,E,F). Two unpaired blood vessels enter the median protocerebrum from the posteroventral direction, uniting into a single blood vessel which leaves the median protocerebrum dorsally (not shown). The olfactory lobe (ON) protrudes laterally from the rest of the deutocerebrum (II; Figures 6A-D,H,I, 7C,E-G). The median protocerebrum (Ic) lies anterodorsal to the deutocerebrum (II; Figures 6D, 7C). The neuraxis flattens posterior to the deutocerebrum (II) and takes on the anteroposterior course of the ventral nerve cord (III, MdG; Figures 6D, 7C).

#### Soma clusters

The brain of *S. lepidops* features five pairs of soma clusters and one unpaired soma cluster (i.e., altogether eleven soma clusters).

The unpaired soma cluster consists of anteroventral somata (AvS) that lie ventrally in the median protocerebrum (Ic; Figures 6F-I, 7A-D). On each side, it has a posterior extension reaching along the ventral and medial side of the deutocerebrum (II; Figures 6H,I, 7A-D). The anterior and posterior regions of the median protocerebrum lack somata.

The first paired soma cluster consists of anterolateral somata (AlS, arrowheads), surrounding the neuropil of the distal protocerebral neuropil (Ia; Figures 6E, 7A,C) and covering the anterior and lateral region of the lateral protocerebrum (Ib; Figure 7A-D). Distally, this soma cluster extends far into the eyestalks (St; Figure 6E).

Also the second paired soma cluster consists of anterolateral somata (AlS, arrows), but it lies apart from the previous one, dorsally in the lateral protocerebrum (Ib; Figure 7A-D). The anterodorsal and posterior regions of the lateral protocerebrum as well as the protocerebral tracts are not covered by somata.

The third paired soma cluster consists of anterodorsal somata (AdS) that are situated dorsally and dorsolaterally in the median protocerebrum (Ic; Figures 6F-H, 7A-D). Each cluster shows a long dorsal extension and may be connected to its counterpart by a thin midline-spanning bridge consisting of only a few somata (AdS; Figures 6F, 7A,B).

The fourth paired soma cluster consists of lateral somata (LS) situated dorsally, laterally, and posteriorly in the olfactory lobes (ON; Figures 6H,I, 7A-D) and dorsolaterally in the rest of the deutocerebrum (II; Figure 7A-D). Also this cluster has a long dorsal extension (LS; Figures 6H, 7A-D). Although it is here described as a separate soma cluster, thin extensions of only a few somata may connect it to neighboring soma clusters, respectively. The medial sides of the deutocerebrum lack somata.

The fifth paired soma cluster consists of posterior somata (PS) that lie posterodorsally in the tritocerebrum (III), opposite to the root of the antenna 2 nerve (A2Nv; Figures 6J-L, 7A,C). It has a long extension projecting posterodorsally (PS; Figures 6L, 7A-D).

In addition to the eleven large soma clusters in the periphery of the brain, several single somata are scattered within the neuropil of the median protocerebrum (Ic; see Figure 6F,G).

#### Neuropils

##### Distal protocerebral neuropil (Ia)

The distal protocerebral neuropil is a small and distinct spheroidal neuropil, which is uniformly structured and directly borders the lateral protocerebrum (Ib; Figures 6B-E, 7F-H). It is directed towards the tip of the eyestalk (St; Figure 6E).

##### Lateral protocerebrum (Ib)

The terminal medulla is half as long as the eyestalk, shows an ellipsoid shape, and constitutes the only neuropil in the lateral protocerebrum of *S. lepidops* (TM; Figures 6A-E, 7A,C,E-H).

##### Median protocerebrum (Ic)

The central body (CB) appears as a densely textured, spindle-shaped neuropil that lies horizontally across the center of the median protocerebrum (Ic; Figures 6F,G, 7E-H). It is anteriorly subdivided into several small vertical lamellae (Figure 6F). The protocerebral bridge (PB) is composed of a pair of elongated neuropils lying horizontally at the dorsal end of the median protocerebrum that are connected across the midline via a thin tract (Ic; Figures 6F, 7E-H). On each body side, an elongated ventromedial protocerebral neuropil (VmPN) extends diagonally through the ventromedial region of the median protocerebrum (Ic; Figures 6F,G, 7E,F,H). Anteriorly, the neuropil fuses with its surroundings. Its posterior end lies anterior to the root of the antenna 1 nerve (A1Nv; Figure 7H). The lateral accessory lobe (LAL) lies laterally in the median protocerebrum, lateral to the central body (Ic; Figures 6F,G, 7E-H). Its texture is denser than the surrounding neuropil.

##### Deutocerebrum (II)

With respect to the size of the brain, the olfactory lobe (ON) in *S. lepidops* is comparably smaller than that in *M. halope*. The olfactory lobe in *S. lepidops* shows a nearly spheroidal shape and is composed of numerous spheroidal olfactory glomeruli (ON, points; Figure 6H,I). The unpaired median antenna 1 neuropil (MAN) spans across the midline of the deutocerebrum (II; Figures 6H, 7E-H). Its lateral end is slightly bent posteriorly (MAN; Figure 7G). The lateral antenna 1 neuropil (LAN) lies in the dorsal region of the deutocerebrum (II; Figures 6I, 7E,G,H), close to the olfactory lobe (ON; Figure 6I).

##### Tritocerebrum (III)

The ventral tritocerebral neuropil (VTN) lies ventrally in the tritocerebrum (III; Figures 6K, 7E-H), directly posterior to the root of the labral nerve (LbNv; Figures 6K, 7H).

#### Tracts

The protocerebral tract (PT) extends from the dorsal region of the lateral protocerebrum (Ib) to the anterolateral region of the median protocerebrum (Ic; Figures 6A-D, 7C,E,F). The olfactory globular tract (OGT) connects the olfactory lobe (ON) to the lateral protocerebrum (Ib), traveling on its way through the median protocerebrum (Ic; Figure 7E-G) and the protocerebral tract (PT). The olfactory globular tracts of both sides form a chiasm posterior to the central body (CB; Figures 6G, 7E,G). The ventral portion of each olfactory globular tract pervades the ventromedial protocerebral neuropil (VmPN; Figures 6G, 7E). One soma-free tritocerebral commissure (TC) arises from the tritocerebrum dorsomedially. The commissure performs a posterodorsal arc when interconnecting both halves of the tritocerebrum transversely (Figures 6A,C,D,L, 7A,E,G,H). On each side, a posttritocerebral connective connects the tritocerebrum (III) to the mandibular ganglion (MdG; Figures 6A,C,D, 7A,C). It is dorsoventrally flattened like the tritocerebrum (Figures 6L, 7E).

The 1^st^ vertical tract (VT1) connects the median protocerebrum with the deutocerebrum and is situated posterior to the lateral accessory lobe (LAL; Figure 7E,G,H). The 2^nd^ vertical tract (VT2) spans between the dorsal and ventral region of the median protocerebrum (Ic), and lies anterior to the central body (CB; Figure 7F,H). The (vertical) Y/Z, the X, and W tracts arise from the dorsal side of the central body and extend dorsally towards the protocerebral bridge. The Y/Z and X tracts pass the olfactory globular tract anteriorly and were traced as far as the protocerebral bridge. In contrast, the W tract passes the olfactory globular tract posteriorly, and is not associated with the protocerebral bridge (Figure 7E-G). One neurite bundle from the dorsal portion of the W tract was traced into the anterodorsal somata (not shown). The 4^th^ transverse tract (TT4) interconnects the anterodorsal regions of the deutocerebrum (II; Figures 6H, 7E-G). The 5^th^ transverse tract (TT5) extends across the whole median protocerebrum, thereby forming a ventral arc and passing the central body ventrally (CB; Figure 7E-H). The end of the 3^rd^ transverse tract lies close to the arising protocerebral tract. The 6^th^ transverse tract (TT6) lies posteroventral to the 3^rd^ transverse tract and interconnects the lateral accessory lobes (LAL; Figures 6G, 7E,H). The 7^th^ transverse tract (TT7) interconnects the anterior regions of the deutocerebrum and is situated directly anterior to the median antenna 1 neuropil (MAN; Figure 7F). The 1^st^ longitudinal tract (LT1) connects the anterior region of the median protocerebrum with the anterior region of the deutocerebrum. At the midpoint of its way through the ventral region of the median protocerebrum, the 1^st^ longitudinal tract (LT1) leaves the surrounding neuropil, so that its middle portion is only surrounded by anteroventral somata (Figure 6G). On each side, an 8^th^ transverse tract (TT8) connects the nerve root of the tegumentary nerve to the medial region of the tritocerebrum. On its way, the tract remains near the surface of the tritocerebrum, before turning ventrally inside (III; Figure 6J).

#### Nerves

The antenna 1 nerves (A1Nv) enter the deutocerebrum (II) from the anteroventral direction (Figures 6B,D,F,G, 7C). Proximally, each nerve splits into a thick medial and a thin lateral root. The lateral root (A1Nv, rocket) proceeds into the center of the olfactory lobe (ON; Figure 6F), while the medial root enters the rest of the deutocerebrum. Distally, each antenna 1 nerve gives rise to three smaller branches (A1Nv; Figure 6B). The prominent antenna 2 nerve (A2Nv) enters the tritocerebrum (III) from the ventrolateral direction (Figures 6B,C,D,J, 7C,E). After giving rise to the lateral tritocerebral nerve (LTNv; Figures 6A-D, 7C) and tegumentary nerve, the antenna 2 nerve splits distally into two branches that extend into the antenna 2. The tegumentary nerve splits distally into two branches which extend to the inner body wall (Figures 6A-D, 7A,C). The posterodorsal nerve (PdNv) enters the dorsal region of the tritocerebrum (III) from the posterodorsal direction (Figures 6A,C,D, 7A,C). The labral nerve (LbNv) enters the anteroventral side of the tritocerebrum (III) from the anterior direction (Figures 6A,B,D,K, 7C). Distally, it extends into the labrum which lies anteroventral to the tritocerebrum in *S. lepidops*. On each side, a stomatogastric nerve (StNv) arises from the tritocerebrum, anteromedially (Figure 6A-D). The stomatogastric nerves from both body sides (StNv) unite forming a single unpaired nerve in front of the esophagus. This single nerve extends dorsally along the upper side of the gut (Figures 6A,C, 7A).

### *Tethysbaena argentarii* (Thermosbaenacea)

#### General aspects

*T. argentarii* lacks compound eyes and eyestalks (Figure 1D, E). Antenna 1 exhibits a 3-segmented peduncle bearing an up to 5-segmented outer and an up to 10-segmented inner flagellum ([57]; see also Figure 1D, E). Unlike in the other species, the up to nine-segmented antenna 2 of *T. argentarii* is considerably less prominent than antenna 1 ([57]; see also Figure 1D, E). Simple setae are loosely distributed over antenna 1 and 2. In addition, the peduncle of antenna 1 is equipped with feather-like (setulated) setae [57].

**Figure 8:**
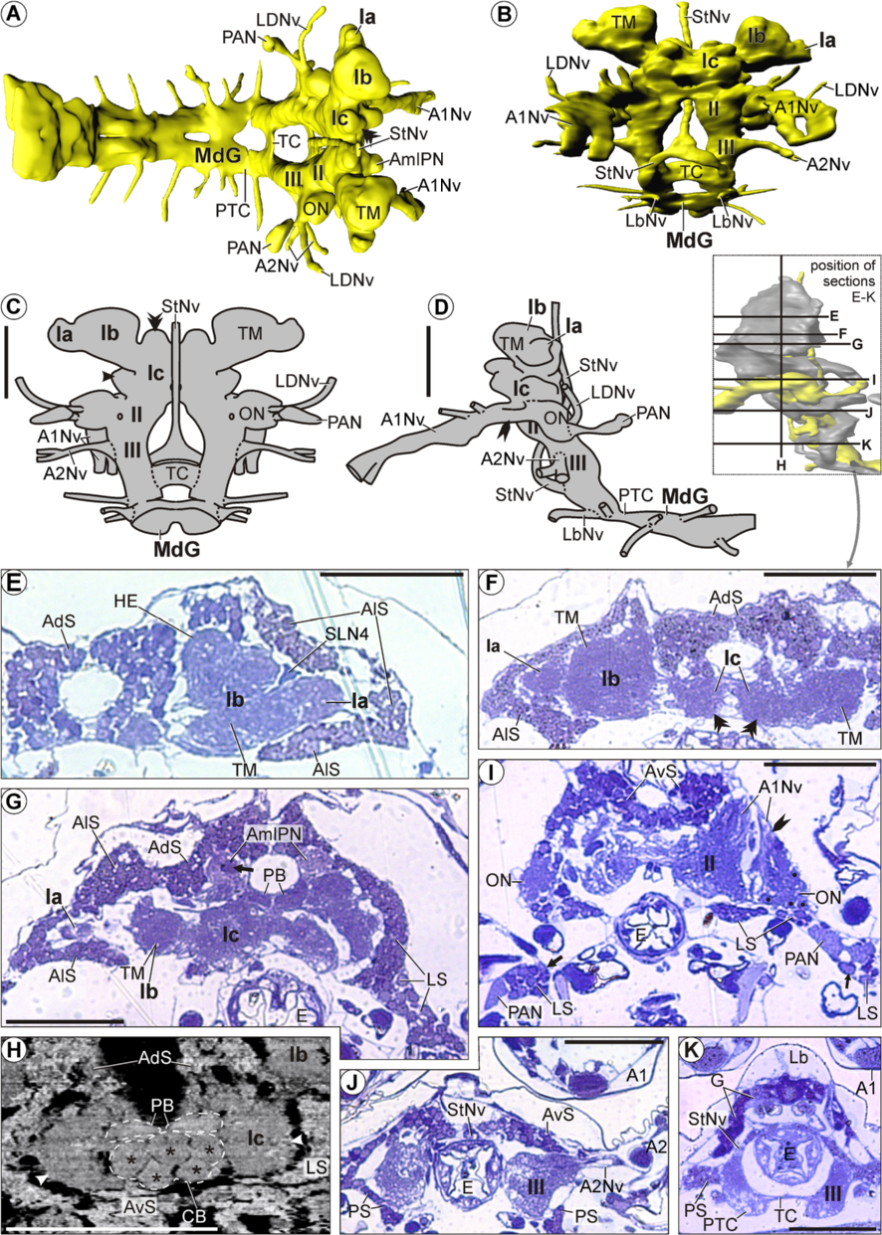
**Morphology of the brain in** ***Tethysbaena argentarii*** **.** Overview and semi-thin sections. **A-D**: Neuropil and nerves without somata. **A**: 3D-reconstruction in dorsal view. Anterior directed towards the right. **B**: 3D-reconstruction in anterior view. **C**: Schematic drawing in posterior view. Double arrowhead marks a dorsal extension, simple arrowhead marks a lateral extension of the median protocerebrum (Ic). **D**: Schematic drawing in lateral view. Rocket marks the lateral root of the antenna 1 nerve (A1Nv). **E**-**G**, **I**-**K**: Horizontal semi-thin sections, ordered from dorsal to ventral. **F**: Double arrowheads as in **A**. **G**: Arrow points at a condensation of neuropil within the anteromedial protocerebral neuropil (AmlPN) that was observed on each body side. **H**: Virtual transverse section. Dotted lines mark the protocerebral bridge (PB) and the central body (CB). The latter is divided into spheroidal subunits (asterisks). Arrowheads as in **A**. **I**: Rocket points at the lateral root of the antenna 1 nerve (A1Nv). Points mark the (only faintly recognizable) olfactory glomeruli in the olfactory lobes (ON). Arrows mark the large posterolateral extensions of lateral somata (LS), which embrace the posterior accessory neuropils (PAN). Scale bars: 50 μm.

**Figure 9:**
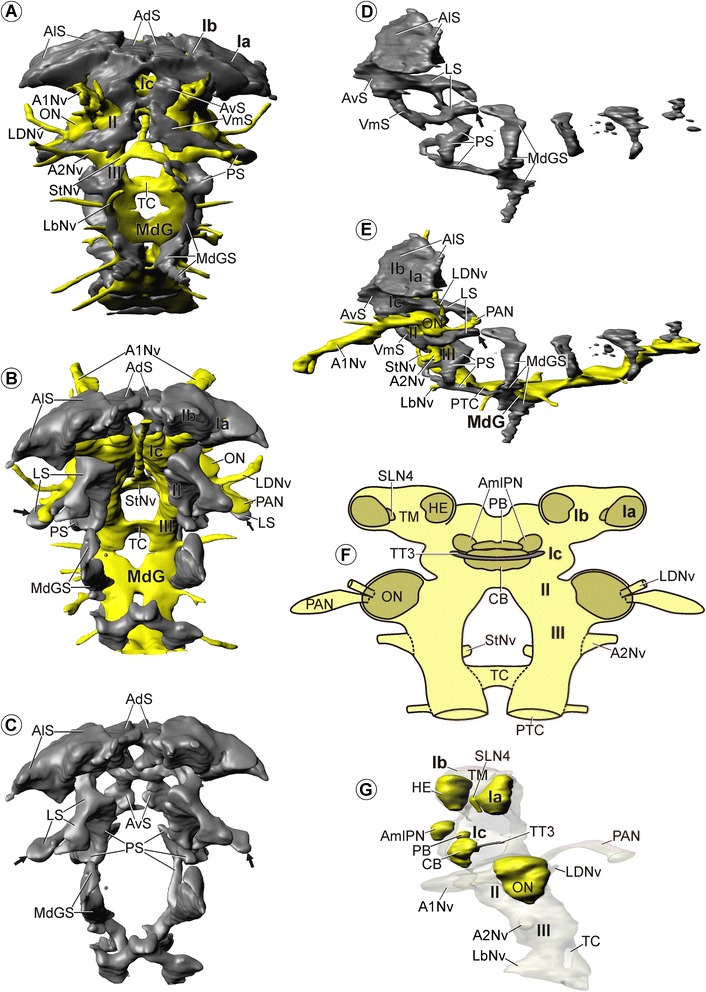
**Morphology of the brain in** ***Tethysbaena argentarii*** **.** Soma clusters, neuropil, and internal structure – click on A and G for interactive 3D models. **A**: Soma clusters (grey) and neuropil (yellow) in anteroventral view. **B**, **C**: Soma clusters in posterodorsal view **(B)**: with and **(C)**: without neuropil. **D**, **E**: Soma clusters in lateral view **(D)**: without and **(E)**: with neuropil. **A**-**E**: Arrows mark the large posterolateral extensions of lateral somata (LS), which embrace the posterior accessory neuropils (PAN, only depicted in **B** and **E**). **F**: Schematic drawing of neuropils (dark yellow) and tracts (grey) in posterior view. **G**: 3D-reconstructions of neuropils and tracts in lateral view. Neuropils are in yellow; tracts are in grey. Brain width about 200 μm.

The distal protocerebral neuropil (Ia) is situated directly lateral to the lateral protocerebrum (Ib; Figure 8A-F). The lateral protocerebrum is directly connected to the median protocerebrum (Ic) ventromedially (Figure 8A-D,F,G). On each side, the neuropil of the median protocerebrum shows a dorsal, an anterior, and a lateral bulge (Figure 8C,F). The median protocerebrum (Ic) lies dorsal to the deutocerebrum (II; Figure 8A-D). The olfactory lobe (ON) protrudes laterally from the rest of the deutocerebrum (II; Figures 8A,C,D,I, 9A,E,F). *T. argentarii* features a posterior accessory neuropil on each side (PAN), which is connected to the deutocerebrum via a thin tract whose target is unclear (Figures 8A,C,D,I, 9B,E,F). The deutocerebrum (II) lies anterodorsal to the tritocerebrum (III; Figure 8A-D). Posterior to the latter, the neuraxis takes on the anteroposterior course of the ventral nerve cord, parallel to the body axis (Figures 8D, 9E).

#### Soma clusters

*T. argentarii* features one pair of soma clusters and one unpaired soma cluster (i.e., altogether three soma clusters).

The paired soma cluster is composed of all anterolateral somata (AlS), covering the distal protocerebral neuropil (Ia) and the lateral protocerebrum (Ib) almost completely (Figure 9A-E).

All other somata of the brain are included in one large unpaired soma cluster. It is situated dorsally (AdS; Figures 8E-H, 9A-C), anteriorly (AvS; Figures 8H, 9A,D,E), and posterolaterally (LS; Figures 8H, 9B-E) in the median protocerebrum (Ic); anteriorly (VmS; Figure 9A,D,E), laterally, and posterolaterally (LS; Figures 8H,I, 9B-E) in the deutocerebrum (II); and laterally and ventrally (PS) in the tritocerebrum (III; Figures 8J,K, 9A-E). A thin row along each posttritocerebral connective even connects the unpaired soma cluster of the brain with the somata of the mandibular ganglion (MdGS; Figure 9D,E). On each body side, the unpaired soma cluster of the brain shows one posterior extension pointing posterolaterally from the median protocerebrum (LS; Ic; Figure 8G) and one posterolateral extension (arrows, LS) pointing posterolaterally and lying adjacent to the posterior accessory neuropil (PAN; Figures 8I, 9B-E). The posterior side of the median protocerebrum (Figures 8F,G, 9B), the lateral side of the olfactory lobe (ON; Figures 8I, 9E), the anterolateral and medial side of the deutocerebrum (II; Figures 8I, 9A,B), and the medial and posterior side of the tritocerebrum (III; Figures 8J,K, 9B) are soma-free.

#### Neuropils

##### Distal protocerebral neuropil (Ia)

The distal protocerebral neuropil (Ia) is a uniformly structured neuropil with a spheroidal shape (Figure 8A-G) and in part confluent with the lateral protocerebrum (Ib; Figure 8E).

##### Lateral protocerebrum (Ib)

The largest region of the lateral protocerebrum is constituted by the terminal medulla (TM; Figures 8E-G, 9F,G). The hemiellipsoid body (HE) is situated anteromedially in the lateral protocerebrum (Ib; Figures 8E, 9F,G). The anteromedial (distal) region of each hemiellipsoid body has a convex shape and is more densely textured than the rest of the hemiellipsoid body. Posterolaterally (proximally), the hemiellipsoid body (HE) is confluent with the terminal medulla (TM; Figure 8E). Furthermore, a glomerular small lateral neuropil (SLN4) is distinguishable directly anterolateral to each terminal medulla (TM; Figures 8E, 9F,G).

##### Median protocerebrum (Ic)

An anteromedial protocerebral neuropil (AmlPN) protrudes anteriorly from the median protocerebrum on each body side (Ic; Figures 8A,G, 9F,G). The medial region (arrow) of each anteromedial protocerebral neuropil (AmlPN) shows a significantly higher neuropil density than the surrounding neuropil (Figure 8G). The unpaired central body (CB) lies horizontally across the center of the median protocerebrum (Ic; Figures 8H, 9F,G). The central body (CB) of *T. argentarii* is comparatively thick and shows a compartmentalization into altogether five spheroidal subunits (asterisks, Figure 8H). The protocerebral bridge (PB; Figure 8G,H) lies dorsally in the median protocerebrum (Ic), adjacent and directly dorsal to the central body (CB; Figures 8H, 9F,G). It is composed of two elongated subunits which are identifiable due to their comparatively dense neuropil texture (Figure 8G,H); these subunits contact one another in the midline. Unlike in *M. halope* and *S. lepidops*, both of which feature a lateral accessory lobe, the median protocerebrum in *T. argentarii* (arrowheads in Figure 8C,H) does not show any compartmentalization or condensation of neuropil in the lateral region.

##### Deutocerebrum (II)

The olfactory lobe (ON) lies lateral to the rest of the deutocerebrum (II), receiving the lateral root of the antenna 1 nerve (A1Nv, rocket) from the anterior direction (Figure 8D,I). It is composed of densely packed olfactory glomeruli (ON, points) whose exact shape could not be identified (Figure 8I). A posterior accessory neuropil is situated posterolateral but slightly distant to each olfactory lobe (PAN; Figures 8A,D,I, 9F,G). The posterior accessory neuropil is embedded within lateral somata that are arranged in a long extension in this region (arrows, LS; Figures 8I, 9B-E). Since the tracts connecting the posterior accessory neuropil to the rest of the brain could not be traced through this group of lateral somata, it remains unclear whether the posterior accessory neuropil is associated with the olfactory lobe or with another region of the deutocerebrum. The texture of the posterolateral accessory neuropil is unstructured (PAN; Figure 8I). Further neuropils could not be distinguished within the deutocerebrum.

##### Tritocerebrum (III)

Distinct neuropils could not be identified in the tritocerebrum in *T. argentarii*.

#### Tracts

The 3^rd^ transverse tract (TT3) is situated posterior to the central body and interconnects the lateral regions of the median protocerebrum (Ic; Figure 9F,G). The tritocerebral commissure (TC) interconnects the halves of the tritocerebrum (III) horizontally (Figures 8A-C,K, 9A,B,F,G). It is anteroposteriorly flattened and has a minute anteroposterior hole in the midline. On each body side, a posttritocerebral connective (PTC) connects the tritocerebrum (III) to the mandibular ganglion (MdG; Figure 8A,D,K). Each posttritocerebral connective is dorsoventrally flattened and medially engraved by a large unpaired transverse apodeme that is associated to several mandible muscles and passes on the dorsal side of the connective.

#### Nerves

The prominent antenna 1 nerve (A1Nv) enters the deutocerebrum (II) from the anterior direction (Figures 8B,D,I, 9A,E), splitting proximally into a thick medial and a thin lateral root. The lateral root (rocket, A1Nv) proceeds into the center of the olfactory lobe (ON), while the medial root proceeds into the rest of the deutocerebrum (Figure 8I). Distally, two smaller branches diverge from each antenna 1 nerve (A1Nv; Figure 8D). A lateral deutocerebral nerve (LDNv) enters the posterior side of each olfactory lobe (ON) from the lateral direction (Figures 8A,C,D, 9A,B,E). The antenna 2 nerve (A2Nv), which is thinner than the antenna 1 nerve in *T. argentarii*, enters the tritocerebrum (III) from the lateral direction (Figures 8B,D,J, 9A,E,G). A stomatogastric nerve (StNv) enters each half of the tritocerebrum (III) from the anteromedial direction (Figure 8B,D,K). Distally, stomatogastric nerves from both body sides unite forming a single unpaired nerve (StNv) in front of the esophagus; this single nerve extends dorsally along the upper side of the gut (E, Figures 8A-D,J, 9A,B). The labral nerve (LbNv) enters the ventral side of the tritocerebrum (III) from the anterior direction (Figure 8B,D).

## Discussion

The general anatomy of the brain in the three investigated species, *Mictocaris halope*, *Spelaeogriphus lepidops*, and *Tethysbaena argentarii*, widely corresponds to the organization in other malacostracans, including the division of the protocerebrum into the subunits of lateral and median protocerebrum; the presence of deutocerebral olfactory lobes; and the location of major nerve roots (e.g., [10,11,22,24,31,55,58]). Although MST show only a few soma clusters, which obscures a comparison with the numerous soma clusters in Decapoda [55], the overall location of soma clusters in the brain is similar. In the following, we focus on morphological structures that have played a role in the debate on phylogenetic and evolutionary relationships.

### Dislocation of brain parts from the eyestalk into the cephalic capsule

An eyestalk housing both the optic lobe and lateral protocerebrum, corresponding to the condition here described for the distal protocerebral neuropil and lateral protocerebrum in *S. lepidops*, occurs also in other taxa ([22]: Leptostraca; [23]: Stomatopoda, Decapoda; [11]: Anaspidacea, Mysidacea; [59]: Euphausiacea) and likely represents the malacostracan ground pattern (see also [22]). The brain parts were dislocated from the eyestalk into the cephalic capsule independently in several blind decapods [10] and *M. halope* (this study).

### Problematic identity of the distal protocerebral neuropils (Ia)

In the malacostracan ground pattern, each optic lobe comprises three comparably large optic neuropils, termed the lamina, medulla (or external medulla), and lobula (or internal medulla), and an additional fourth, smaller optic neuropil termed the lobula plate, which has only been described in some taxa [22,30,60]. Apparently, in any case, a reduction of the optic lobes has occurred in MST, which exhibit only one distal protocerebral neuropil. In order to infer its evolutionary identity, the distal protocerebral neuropil has to be compared to neuropils and neuropil domains in the protocerebrum of other taxa.

#### Comparison to optic neuropils

In Leptostraca, Decapoda and Isopoda, the four optic neuropils can be identified and homologized on the basis of their interconnecting decussating and non-decussating tracts (see [30]), but comparable tracts were not identified in the small representatives of MST. In *S. lepidops*, the distal protocerebral neuropil is most plausibly interpreted as a single optic neuropil, since it is directed towards the tip of the eyestalk, as are the optic neuropils in Decapoda [19-22,30,43], Stomatopoda [10,43] or Euphausiacea [59]. Also in *M. halope* and *T. argentarii*, the distal protocerebral neuropil could be interpreted as a single optic neuropil. Yet this would not be supported by the position of the distal protocerebral neuropil, which lies laterally in the lateral protocerebrum, while the eyestalk in *M. halope* inserts much more anteriorly. With its spheroidal shape and even texture, the distal protocerebral neuropil in MST corresponds most closely to the lobula in other Malacostraca, but differs from the lamina and medulla, which are distally convex and flattened and exhibit a visibly columnar retinotopic texture of neuropil (see, e.g., [19,20,59]). The main function of the lobula in other malacostracans, and also in insects, lies within motion detection of compound eye input [43], which is certainly not the case in the eyeless representatives of MST. The lobula plate of other malacostracans [30,43,60] is, due to its small size, an unlikely candidate for homology with the distal protocerebral neuropil of MST.

Although size-reduction of the optic lobe has occured several times in Eumalacostraca, e.g., within Brachyura and Anomala [10], the most drastic reductions have obviously occurred within Peracarida. Hitherto, the representatives of MST (this study) are the only reported eumalacostracans which have lost (at least) two optic neuropils completely. Unpublished investigations (MEJS, SR, CSW) revealed that the blind cumacean *Leucon nasica* also lacks a lamina and a medulla, and that the tanaidacean *Tanais dulongi* with its rudimentary compound eyes lacks a lamina, but still features a small medulla and a lobula. Blind non-malacostracan crustaceans lacking both compound eyes and optic neuropils are Cephalocarida [54,61], Remipedia [62], and Mystacocarida [63]. Reductions or loss of visual brain centers are also common in blind representatives of other metazoan groups (reviewed by [64]).

#### Comparison to subunits of the lateral protocerebrum

An alternative interpretation of the identity of the distal protocerebral neuropil applies to *M. halope* and *T. argentarii.* In both species, the far-lateral position of the neuropil supports that it constitutes a lateral subunit of the terminal medulla. This counts also for the blind cumacean *Leucon nasica*, which exhibits a corresponding distal protocerebral neuropil posterolaterally in the terminal medulla, and which lacks optic neuropils (unpublished data by MEJS, SR, CSW). All optic neuropils would have been reduced completely in *M. halope*, *T. argentarii,* and *L. nasica*. Although Kenning et al. [22] have not reconstructed a comparable distal protocerebral neuropil in the malacostracan ground pattern, several other authors have described the terminal medulla in Stomatopoda, Decapoda, and Isopoda as an unspecific aggregation of structured and unstructured neuropils e.g., [11,23,65]. Also in Remipedia, a possible sister-group to Malacostraca [15], the protocerebral neuropil is laterally compartmentalized into sublobes, some of which correspond in position to the distal protocerebral neuropil [66,67]. In our view, the identity of the distal protocerebral neuropil in *M. halope* and *T. argentarii* remains unresolved. It could either represent an optic neuropil or a lateral subunit of the terminal medulla. Other interpretations are unlikely: Neither is the distal protocerebral neuropil homologous with the pronounced domain of pigment dispersing hormone (PDH) immunoreactivity in Decapoda, which is situated medially in the terminal medulla, receives axons from the optic neuropils, and has been suggested to function as a pacemaker of circadian clocks [68]. No PDH immunolabeling has been conducted in MST or any other eyeless arthropod, and information on circadian rhythms in blind arthropods is generally scarce (reviewed by [69]). Nor is the distal protocerebral neuropil homologous with the hemiellipsoid body, which is situated elsewhere, i.e., anteromedially or anteriorly in the lateral protocerebrum (Malacostraca: [22,32,65]; Remipedia: [66]; see below).

### Unclear identity of the small lateral neuropils

Two small lateral neuropils comparable to those in *M. halope* and *T. argentarii* occur in the lateral protocerebrum of Leptostraca (labeled sN and X by [22]). Kenning et al. [22] homologized one with the eumalacostracan lobula plate. The position of the small lateral neuropils is different between Leptostraca and those in *M. halope* and *T. argentarii*, but we found three small lateral neuropils in the cumacean *Leucon nasica* (unpublished data by MEJS, SR, CSW)*,* which correspond exactly to *M. halope*.

### Central complex corresponds in detail to Decapoda

One of the most interesting structures in the tetraconate brain is certainly the central complex, an intricate cluster of two unpaired and one pair of neuropils (central body, protocerebral bridge; lateral accessory lobes) and their interconnecting tracts (reviewed by [29]: Crustacea; [70]: Hexapoda). The protocerebral bridge and central body have been described in most malacostracan subtaxa including MST (this study), Leptostraca [22], Stomatopoda [11], Decapoda [20,21,71], Euphausiacea, Anaspidacea [24], Isopoda [32,72]. Four conspicuous W, X, Y, Z tracts connect the protocerebral bridge with the central body on each side in Decapoda (e.g., [71]), Isopoda, Cumacea [29], and Leptostraca, a pattern which dates back to the ur-malacostracan [22] and has similarly been found in Remipedia [67] and Hexapoda (e.g., [73]). The lack of distinct W, X, Y, Z tracts in *M. halope* and *T. argentarii* and the reduced number of only three tracts in *S. lepidops* are here interpreted as derived. The lateralmost tract in *S. lepidops* is the only tract posterior to the olfactory globular tract, corresponding to the W tract in other malacostracans (e.g., [71]). Our interpretation of the other tracts in *S. lepidops* remains ambiguous. The two medialmost Z and Y tracts in Isopoda are spatially close (see Figure 2A in [29]), which might indicate that the single medialmost tract in *S. lepidops* is a product of fusion.

Since the subunits in *T. argentarii* are larger than in *M. halope*, considerable doubt remains on whether the spheroidal differentiation is homologous. In contrast, the vertical differentiation of the central body in *S. lepidops* corresponds to the *“*almost columnar appearance” in different Decapoda [71,74], Isopoda, Stomatopoda [43] and also Hexapoda (e.g., [43,44,75]).

The central complex in Hexapoda functions as a second order visual center, but also plays a major role in spatial learning, spatial memory, and in the integration of spatial information for locomotor control of walking and flight (reviewed by [70]). Several blind arthropods exhibit well-developed unpaired midline neuropils, such as Remipedia with their large protocerebral bridge [62,67] or Symphyla [9]. With *S. lepidops* and *M. halope*, we add two other species to this group.

### Olfactory system

#### Olfactory lobe

The relatively large size of the olfactory lobe in *M. halope* and *S. lepidops* (this study) co-occurs with a lack of eyes and reduction of optic neuropils, such as in other blind crustaceans like Cephalocarida [54] and Remipedia [67]. Yet an enlargement of the olfactory lobe is not necessarily the case in blind crustaceans. A comparably small olfactory lobe occurs in *T. argentarii* (this study) and the blind cumaceans *Leucon nasica* (unpublished data by MEJS, SR, CSW) and *Diastylis rathkei* ([28]; see Figure 2C in [29]). In contrast, a large olfactory lobe occurs along with large optic neuropils in diverse malacostracans (e.g., [32]: Isopoda; [19-21]: Decapoda).

The spheroidal shape of olfactory glomeruli in *M. halope* and *S. lepidops* represents the malacostracan [17,22] and mandibulate ground pattern [53,54] and differs from the derived elongate or discoidal shapes of glomeruli in Decapoda [19-21,76], Cephalocarida [54], and Chilopoda [53].

The elongated evenly-textured posterior accessory neuropil, which is set off the olfactory lobe in *T. argentarii* (this study), differs from the more compact glomerular accessory lobe that is associated with the olfactory lobe in Reptantia (e.g., [17,19,20,58]). Given their phylogenetic position [8], both structures must have evolved convergently. Whether the posterior accessory lobe constitutes an analogous olfactory center remains unclear, as its offgoing tracts were hard to trace.

#### Hemiellipsoid body

In the lateral protocerebrum of Stomatopoda and Decapoda, each olfactory globular tract splits to innervate the terminal medulla and hemiellipsoid body, both of which were suggested to function as second order olfactory centers [23,77]. Although the hemiellipsoid body in *T. argentarii* is relatively small, it corresponds to the hemiellipsoid body in other malacostracans in (1) its medial position in the protocerebrum, (2) its distally densified texture, and (3) its distally convex neuropil (compare [22]: Leptostraca; [11,24]: Euphausiacea, Anaspidacea; [19-21,78]: Decapoda; [10,11]: Mysida).

In contrast, a hemiellipsoid body is absent in *M. halope*, *S. lepidops* and many other peracarids ([27]: Tanaidacea; [28]: Cumacea; [11]: Amphipoda; [31]: Isopoda) – irrespectively of whether these taxa exhibit a small or large olfactory lobe. As revealed by own investigations on the basis of semi-thin sections (unpublished data by MEJS, CSW, SR), a hemiellipsoid body is also absent in *Leucon nasica* (Cumacea), *Paramphisopus palustris* (Isopoda), *Tanais dulongi* (Tanaidacea), and *Hyalella azteca* (Amphipoda), but present in the anteromedial region of the lateral protocerebrum of *Lophogaster typicus* (Lophogastrida). It is interesting that most taxa lacking a hemiellipsoid body belong to a ‘core group’ of Peracarida that appeared monophyletic in several independent analyses (Amphipoda + Mancoida *sensu lato*, see [8]), so that the reduction of the hemiellipsoid body might represent an apomorphy. However, this hypothesis is challenged by recent descriptions of a hemiellipsoid body in the isopods *Saduria entomon* ([32]: stained synapsin) and *Idotea emarginata* ([65]: lipophilic dye fills).

Since *M. halope* and *S. lepidops* are blind and, given their large olfactory lobe, seem to depend largely on the olfactory sense, we may conclude in line with Sullivan and Beltz [23] that their widely undifferentiated terminal medulla has taken over the role of a second order olfactory center. The bifurcation of the olfactory globular tract in *M. halope* (Figure 4E) is reminiscent of that in other malacostracans, whose olfactory globular tract splits to innervate the terminal medulla and hemiellipsoid body [11,23,65,77,79,80].

### Mechanosensory neuropils in the deuto- and tritocerebrum

The mechanosensilla, statocysts, and non-olfactory chemoreceptors on the antenna 1 of Decapoda send their afferents into the deutocerebral ‘lateral antenna 1 neuropil’ [81-87]. A corresponding neuropil occurs in Leptostraca [22], Stomatopoda [88], Mysidacea [26], Euphausiacea [58], Anaspidacea [24], Isopoda [21,32], and also in *M. halope* and *S. lepidops* (this study). In all these malacostracans and other crustaceans such as Remipedia [67] or Cephalocarida [54], the antenna 1 nerve splits proximally into a lateral root proceeding into the olfactory lobe and a medial root proceeding into the lateral antenna 1 neuropil, implying a non-olfactory function of the latter.

As discussed by Kenning et al. [22], also the unpaired ‘median antenna 1 neuropil’ in Decapoda receives primary afferents from the proximal antenna 2 segments, in particular from the statocysts (e.g., [81,82,84]). Facing the “bilaterally symmetrical” organization of this neuropil in Leptostraca [22], Stomatopoda [88] and Remipedia [66], Kenning et al. [22] assigned a paired ‘median antenna 1 neuropil’ to the ground pattern of Malacostraca. The paired median antenna 1 neuropil in *M. halope* would be plesiomorphic to this pattern, the unpaired one in *S. lepidops* (this study) would have fused independently from Decapoda.

Also the tritocerebral ‘antenna 2 neuropil’ and ‘tegumentary neuropil’, which are directly associated with the antenna 2 nerve and tegumentary nerve in diverse Malacostraca, were related to mechanosensory and motory function and assigned to the malacostracan ground pattern [22]. Although a thick antenna 2 nerve and a distinct tegumentary nerve occur also in *M. halope* and *S. lepidops*, their tritocerebral neuropils are either too large and indistinct (LTN) or situated too far ventrally (VTN) to be homologized unambiguously with the mentioned neuropils in other malacostracans. In fact, the ventral tritocerebral neuropil in *M. halope* and *S. lepidops* are more likely to be associated with the labral than with the antenna 2 nerve. A ‘striated’ or ‘microglomerular’ texture of tritocerebral neuropils, as reported in Isopoda [31,32] or Decapoda [21,89] is not apparent in the tritocerebrum of *M. halope* and *S. lepidops*.

### Deutocerebrum and tritocerebrum in the light of sensory ecology

Based on the anatomy of the mouthparts in MST, two modes of feeding have been suggested: Either the animals scrape food particles from a substratum with their maxilla 1, then hold and move these particles with their mandibular palp, maxilla 2, and maxillipede, and finally chew them using their mandibular mola; or the animals bite directly into the substratum using their mandibular incisa and lacinia [37,90]. In any case, food has to be located in the darkness. The presence of a prominent olfactory lobe in *M. halope* and *S. lepidops* conforms well with the fact that the long antenna 1 in both species is equipped with several distal aesthetascs [37,38]. In its cavernous natural habitat, *M. halope* could be observed to swim primarily in midwater, the antennae being directed forward at an angle of about 60° to 90° from each other [37]. Interestingly while walking, the antennae of *M. halope* were held much wider apart at an angle approaching 180° [37], a behavior which seems in our view more suitable to catching floating particles than to touching for substrate. Also *T. argentarii* is able to swim vividly and, under laboratory conditions, the species has been observed to actively choose a vegetable instead of carnal nourishment [91]. This strongly supports that the olfactory lobe and hemi-ellipsoid body are functional despite of their comparatively small size, and that at least some of the described setae on the thermosbaenacean antenna 1 [57] are chemosensory. Apart from olfactory function, the fast swimming movements reported in all the three representatives of MST [37,38,91] and the considerable length of their antenna 1 and 2 (e.g., Figure 1) render likely that the appendages are involved in some sort of mechanosensory function. While the lateral antenna 1 neuropil and median antenna 1 neuropil in *M. halope* and *S. lepidops* are possible candidates for mechanosensory centers in the deutocerebrum, here it remains unclear why the tritocerebral mechanosensory neuropils reported in other malacostracans are absent in MST.

### Ventral nerve cord of *Mictocaris halope*

#### General aspects

The ventral nerve cord has been studied mainly in Decapoda (reviewed in [92,93], see also [10,94-96]). Some data exist also for Leptostraca [10,97,98], Stomatopoda [99], Euphausiacea, Tanaidacea [10], and Isopoda [10,72,100,101]. While the paired connectives in all Malacostraca are separated in the midline, segmental pairs of separated ganglia have only been described in the ventral nerve cord of Isopoda and Tanaidacea [10]. Other Malacostraca including *M. halope* exhibit unpaired segmental ganglia with a medially contiguous soma cortex, respectively [10,94,96,102]. In these taxa, the segmental neuropils are bilaterally separated and interconnected by commissures as in Euphausiacea or Decapoda [10,94-96], or they are medially fused embracing the commissure-like tracts as in *M. halope*. It remains unclear, which of the described patterns of median fusion is apomorphic within Malacostraca. In any case, the rope-ladder-like arrangement of commissure-like tracts and connectives in *M. halope* corresponds to that in other arthropods [56,103].

#### Degree of fusion in the subesophageal ganglion

The degree of fusion between subesophageal neuromeres varies considerably across Malacostraca, which hinders a reconstruction of the ground pattern. Free segmental ganglia connected by soma-free connectives occur in Leptostraca [10,22,97], *S. lepidops*, and *T. argentarii* (this study) and may represent the malacostracan ground pattern. The subesophageal ganglion in Euphausiacea comprises two neuromeres (Md, Mx1, see Figure 448IV in [10]), and in Stomatopoda [99], Decapoda [104], and Isopoda [100], it comprises the three subesophageal neuromeres (Md, Mx1, Mx2) as well as additional neuromeres of the thorax. *M. halope* is the only malacostracan with a subesophageal ganglion composed of exactly three neuromeres (this study). As this pattern has been found in Hexapoda (see [103]), some Myriapoda [105], and Cephalocarida [106], it has been suggested for the mandibulate ground pattern (discussed critically by [106]).

#### The terminal ganglion consists of at least two fused ganglia

The posteriormost ganglion of the malacostracan ventral nerve cord is situated in the sixth pleomere and commonly referred to as the terminal ganglion. Studies in Leptostraca [98], Mysida and Lophogastrida [107,108], Anaspidacea [109], Isopoda [101], or Decapoda (reviewed by [93]) revealed that the size of the terminal ganglion and its number of offgoing nerves exceed that in the pleonic ganglia 1 to 5, implying that the terminal ganglion is composed of two or more neuromeres. Correspondingly, five pairs of nerves arise from the terminal ganglion in *M. halope*, while only one segmental and one intersegmental nerve arise from the free pleonic ganglia 1 to 5, respectively (this study). We conclude that the terminal ganglion in *M. halope* consists of at least two fused neuromeres.

#### Longitudinal neurite bundles

Corresponding to *M. halope*, a pair of lateral longitudinal neurite bundles has been described in several crustaceans ([110]: Stomatopoda; [111]: Decapoda; [112-114]: Isopoda; [115]: Branchiopoda (*Leptodora kindtii*); [63]: Mystacocarida (*Derocheilocaris remanei*); [106]: Cephalocarida). The lateral neurite bundle in Mictocarididae (this study), Mystacocarida [63], and Cephalocarida [116] is associated with the intersegmental nerve, supporting homology of the lateral neurite bundle. Lateral neurite bundles are also known from some representatives of Chelicerata [103,117], indicating that this feature might even date back to the ancestor of all arthropods [63].

## Conclusions

All three studied representatives of MST show a considerable size reduction of the optic lobe in correlation to blindness. Moreover, the number of optic neuropils was reduced from four in the malacostracan ground pattern [22] to one (or none). While the distal protocerebral neuropil in *S. lepidops* is best interpreted as the lobula, an alternative interpretation is possible in *M. halope* and *T. argentarii*. There, the distal protocerebral neuropil either represents the lobula (which would have been dislocated laterally), or it represents a lateral subunit of the terminal medulla. The presence of a distinctive central complex, especially in *S. lepidops* and *M. halope*, adds support to a central coordinating function of this structure, irrespectively of whether its sensory input is visual (see also [43,44,70]). Detailed correspondences in the arrangement of W, X, Y, Z tracts of the central complex between Spelaeogriphacea, Isopoda, Cumacea, Leptostraca and Decapoda support that a central complex was already present in the ancestor of Malacostraca [22,29]. The large olfactory lobe in *M. halope* and *S. lepidops* implies an important role of the olfactory sense. Although *T. argentarii* exhibits only a small olfactory lobe, the unique posterior accessory lobe in the deutocerebrum may play an olfactory role analogously to the accessory lobe of Reptantia. The lack of a hemiellipsoid body in *M. halope* and *S. lepidops* implies that the terminal medulla takes over the role of a second order olfactory center completely. Recent descriptions of a hemiellipsoid body in Isopoda on the basis of different methods [32,65] imply, in our view, that the lack of a hemiellipsoid body in other mancoids (*M. halope*, *S. lepidops*, Cumacea, Tanaidacea), and Amphipoda, has to be explained by several independent steps of reduction. Our finding of a protocerebral split in the olfactory globular tract of *M. halope* is in line with this explanation. Distinct mechanosensory centers are not discernible.

The phylogenetic implications of our data are weak. If MST form a monophylum [7,8], the loss of the lamina and medulla could be interpreted as an apomorphy of this taxon. If alternatively Thermosbaenacea are a sister group to all (other) Peracarida (e.g., [1]), this loss of optic neuropils would require more transformational steps. The independent loss of optic neuropils in other peracarid taxa with reduced or lost eyes (Tanaidacea, Cumacea) raises doubt in the phylogenetic value of this feature. Convincing apomorphies of either a monophyletic MST, or of Mictocarididae + Spelaeogriphacea, have not been found.

## Methods

### Collection data of the studied taxa

One representative species was chosen from each of the three taxa MST, respectively. The specimens of *Mictocaris halope* Bowman and Iliffe, 1985 [37] (Mictocarididae) were collected through scuba dives in Deep Blue Cave, Bermuda by Thomas Iliffe and Deron Long. The specimens of *Spelaeogriphus lepidops* Gordon, 1957 [38] (Spelaeogriphacea) were collected on Table Mountain, South Africa, by Stefan Moser, Stefan Richter, and Christian Wirkner. The specimens of *Tethysbaena argentarii* Stella, 1951 [57] (Thermosbaenacea) were collected in Monte Argentario, Italy, by Carsten Wolff, Stefan Richter, and Christian Wirkner.

### Histological sections

Several histological semi-thin section series were carried out for each species. Therefore, the head was first cut off the animal and fixed in Bouin’s fixative. It was then dehydrated in ethanol and, after an intermediate step of epoxypropane, embedded in araldite epoxy resin under vacuum. Serial semi-thin sections (500 nm or 1 μm) were made with a Leica Ultracut UCT microtome using diamond knives in horizontal and transverse plain, respectively, and for *M. halope* additionally in sagittal plain. The sections were stained with a mixture of 1% azure II and 1% methylene blue in aqueous 1% borax solution for approximately 5-25 s at 80-90°C.

### 3D-modeling

For each species, one of the semi-thin section series was further processed for 3D-reconstruction. Histological sections were digitized with a digital camera (PixaLinkPL A622C for *M. halope* and Zeiss AxioCam ICc 3 for the other species) mounted on a light microscope (Zeiss Axio Scope). Sections were digitized in lower magnification yielding an overall image of the whole brain in relation to other organ systems, and in higher magnification yielding a more detailed image of its substructures. The digitized sections were aligned and combined to a 3D virtual stack using the software AutoAligner by Bitplane. All 3D-reconstructions were performed using the software Imaris versions 4.0.5 through 6.0.2 by Bitplane. Therefore, the contours of an investigated structure were marked with polygons on each digitized section, manually. On the basis of the resulting polygon scaffold, a 3D-model was created by surface rendering. 3D-models were integrated into PDF files using the Deep Exploration 5 software (Right Hemisphere, San Ramon, CA). All figure plates were created and labeled using Corel Draw X3 software. The bitmap images were edited using Corel PhotoPaint X3. Both programs are included in the Corel Graphics Suite X3 software package by Corel.

### Immunocytochemistry

Individuals of *Mictocaris halope* were fixed in 4% paraformaldehyde dissolved in phosphate-buffered saline (PBS, 10 mM sodium phosphate, 150 mM sodium chloride, pH 7.4) directly after collection. As adults from *Mictocaris halope* are only about 3 mm in size, dissection of the nervous system is quite difficult, especially under field conditions. In order to manage the balancing act between a complete preservation of nervous system and well-fixed tissue, we treated the specimens in two ways: two specimens were fixed as a whole without any dissection. In three individuals, the head region together with some trunk segments was cut off and both parts were fixed. After fixation, animals were transferred to PBS with 0.5% sodium azide and stored at 4°C until use.

For vibratome sections, the tissue was covered for a short period in Poly-L-Lysin in order to achieve better connection of tissue and embedding medium. After removing the Poly-L-Lysin, the tissue was embedded in 7% low melting point agarose (Roth) dissolved in aqua dest. at approximately 35°C. After cooling to room temperature, the trimmed blocks were sectioned horizontally into 50 μm thin sections with a Leica VT 1000 S vibratome.

All immunocytochemical steps were performed on a shaker with smooth agitation at room temperature if not otherwise stated. After sectioning, slices were permeabilized for 45 min in 0.3% Saponin in PBS containing 0.3% Triton X-100 (PBS-TX 0.3%). Then, the tissue was washed three times in PBS-TX 0.3%, followed by a blocking step in 5% normal goat serum in PBS-TX 0.3% for 3 h or overnight (4°C). Subsequently, the slices were incubated in the primary antibody mouse anti-acetylated α-tubulin (Sigma, cat. no. T6793, lot no. 059 K4823, clone 6-11B-1) diluted 1:2000 in blocking solution overnight at 4°C. After three washes with PBS-TX 0.3% for 15 min each, the tissue was incubated in the secondary antibody goat anti-mouse Alexa Fluor 488-conjugated (Molecular Probes), diluted 1:250 in blocking solution plus 4’,6-diamidino-2-phenylindole-dihydrochloride (DAPI, 1 μg/ml) for counterstaining the nuclei. Then, the tissue was rinsed twice in PBS-TX 0.3% and once in PBS. The slices were mounted on adhesive glass slides in Mowiol (Roth).

### Antibody specificity

The monoclonal antibody raised against acetylated α-tubulin from the sea urchin *Strongylocentrotus purpuratus* (Sigma, cat. no. T6793, lot no. 059 K4823, clone 6-11B-1) reacts with acetylated α-tubulin over a wide range of organisms such as plant, human, pig, monkey, invertebrates, hamster, bovine, chicken, rat, frog, protista and mouse (see datasheet manufacturer). This antibody was utilized in numerous studies on the nervous system of diverse crustacean taxa (e.g., [118]: Branchiopoda; [54]: Cephalocarida; [119]: Malacostraca; [63]: Mystacocarida; [67,120]: Remipedia). Thus, the recognized epitope seems to be highly conserved across life forms, which leads to the suggestion that the antiserum labels acetylated α-tubulin also in *Mictocaris halope*.

### Confocal microscopy and image processing

The physical sections of 50 μm were viewed with a Leica TCS SP5 confocal laser-scanning microscope using Leica LAS AF software. Optical sections with 0.5 μm thickness were taken from the physical slices. These z-series were processed with NIH ImageJ, v. 1.46r (Rasband WS, ImageJ, U.S. National Institutes of Health, Bethesda, MD, http://imagej.nih.gov/ij/), producing depth coded images and merging channels. The quality was enhanced by adjusting brightness and contrast if necessary and photographs were arranged using Adobe Photoshop 6.0 (San Jose, CA).

## Abbreviations

A1-2: Antenna 1 and 2
A1Nv: Antenna 1 nerve
A2Nv: Antenna 2 nerve
AdS: Anterodorsal somata
AlS: Anterolateral somata
AmlPN: Anteromedial protocerebral neuropil
AmnPN: Anteromedian protocerebral neuropil
AvS: Anteroventral somata
BA: Brain artery
CB: Central body
Con: Connective
E: Esophagus
G: Gland cell
GC: Globular cavity
HE: Hemiellipsoid body
Ia: Distal protocerebral neuropil
Ib: Lateral protocerebrum
Ic: Median protocerebrum
II: Deutocerebrum
III: Tritocerebrum
IsNv: Intersegmental nerve
LAL: Lateral accessory lobe
LAN: Lateral antenna 1 neuropil
Lb: Labrum
LbNv: Labral nerve
LDNv: Lateral deutocerebral nerve
LPNv: Lateral protocerebral nerve
LS: Lateral somata
LT1: 1^st^ longitudinal tract
LTN: Lateral tritocerebral neuropil
LTNv: Lateral tritocerebral nerve
MAN: Median antenna 1 neuropil
Md: Mandible
MdG: Mandibular ganglion
MdGS: Somata of mandibular ganglion
MdNv: Mandibular nerve
MST: Summarizing abbreviation for Mictocarididae, Spelaeogriphacea, and Thermosbaenacea
Mx1Nv: Maxillular nerve
Mx2Nv: Maxillar nerve
MxpG: Maxillipedal ganglion
OGT: Olfactory globular tract
ON: Olfactory lobe
PAN: Posterior accessory neuropil
PB: Protocerebral bridge
PdNv: Posterodorsal nerve
PdPN: Posterodorsal protocerebral neuropil
PlG1-5: 1^st^-5^th^ pleonic ganglion
PS: Posterior somata
PT: Protocerebral tract
PTC: Posttritocerebral connective
PTS: Posttritocerebral somata
SG: Subesophageal ganglion
SGS: Somata of subesophageal ganglion
SLN1-4: 1^st^-4^th^ small lateral neuropils
SNv: Segmental nerve
St: Eyestalk
StNv: Stomatogastric nerve
TC: Tritocerebral commissure
TG: Terminal ganglion
ThG2-8: 2^nd^-8^th^ thoracic ganglion
TL: Thoracic limb
TM: Terminal medulla
TNv: Tegumentary nerve
TT1-8: 1^st^-8^th^ transverse tract
VmPN: Ventromedial protocerebral neuropil
VmS: Ventromedial somata
VT1-2: 1^st^-2^nd^ vertical tracts
VTN: Ventral tritocerebral neuropil
W, X, Y, Z: W, X, Y, Z tracts
X in Fig. 4E: Chiasma of the olfactory globular tracts
